# From illness management to quality of life: rethinking consumer health informatics opportunities for progressive, potentially fatal illnesses

**DOI:** 10.1093/jamia/ocad234

**Published:** 2023-12-22

**Authors:** Marcy G Antonio, Tiffany C Veinot

**Affiliations:** School of Information, University of Michigan, Ann Arbor, MI 48109, United States; School of Health Information Science, University of Victoria, Victoria, BC V8W 2Y2, Canada; School of Information, University of Michigan, Ann Arbor, MI 48109, United States; Department of Health Behavior and Health Education, School of Public Health, University of Michigan, Ann Arbor, MI 48109, United States

**Keywords:** COPD, palliative care, consumer health informatics, mixed methods research, social isolation

## Abstract

**Objectives:**

Investigate how people with chronic obstructive pulmonary disease (COPD)—an example of a progressive, potentially fatal illness—are using digital technologies (DTs) to address illness experiences, outcomes and social connectedness.

**Materials and Methods:**

A transformative mixed methods study was conducted in Canada with people with COPD (*n* = 77) or with a progressive lung condition (*n* = 6). Stage-1 interviews (*n* = 7) informed the stage-2 survey. Survey responses (*n* = 80) facilitated the identification of participants for stage-3 interviews (*n* = 13). The interviews were thematically analyzed. Descriptive statistics were calculated for the survey. The integrative mixed method analysis involved mixing between and across the stages.

**Results:**

Most COPD participants (87.0%) used DTs. However, few participants frequently used DTs to self-manage COPD. People used DTs to seek online information about COPD symptoms and treatments, but lacked tailored information about illness progression. Few expressed interest in using DTs for self- monitoring and tracking. The regular use of DTs for intergenerational connections may facilitate leaving a legacy and passing on traditions and memories. Use of DTs for leisure activities provided opportunities for connecting socially and for respite, reminiscing, distraction and spontaneity.

**Discussion and Conclusion:**

We advocate reconceptualizing consumer health technologies to prioritize quality of life for people with a progressive, potentially fatal illness. “Quality of life informatics” should focus on reducing stigma regarding illness and disability and taboo towards death, improving access to palliative care resources and encouraging experiences to support social, emotional and mental health. For DTs to support people with fatal, progressive illnesses, we must expand informatics strategies to quality of life.

## Background and significance

Chronic obstructive pulmonary disease (COPD) is a progressive, potentially fatal illness that continues to be under-resourced despite being the third leading cause of death worldwide.^1^ COPD is a heterogeneous lung condition marked by shortness of breath and acute breathing events that can be fatal.^2–5^ Social isolation—well recognized among those with COPD—may mean that these “scary” breathing events are unsupported experiences.^6^^,^^7^ Contributing factors to social isolation include decreased social networks as one ages, loss of ability to work due to loss of physical function, and discouragement from spending time within the community due to poor air quality or fear of viral infection.^5^^,^^8–11^ Because COPD is characterized by diminished physical ability and noticeable respiratory difficulties such as coughing and oxygen therapy, there can be stigma concerning disability.^12–14^ Furthermore, with smoking often cited as the greatest “risk factor” for COPD, people may experience stigma and self-blame for having caused their illness.^12^^,^^14–18^ The social isolation and stigmatization of COPD may result in under-treatment of anxiety and depression in the COPD population.^6^^,^^10^^,^^18–20^

Although early diagnosis of COPD and subsequent treatment can delay illness progression and improve patients’ quality of life and mortality rates,^2^^,^^4^^,^^21^^,^^22^ the stigma toward COPD may contribute to inequitable healthcare access and resources.^5^^,^^11^^,^^23–25^ Three quarters of people with COPD may be undiagnosed,^26–28^ and more than a quarter will first learn of their diagnosis upon hospitalization.^25^^,^^29^^,^^30^ Confirmation of diagnosis through spirometry testing^4^^,^^31^ may be delayed, as providers may be uncomfortable about, or not see the need for, testing,^32^ or costs may be prohibitive for underinsured or rural patients.^33^ Although best practice guidelines include attending pulmonary rehabilitation programs,^22^^,^^31^^,^^34^^,^^35^ few people have ongoing access to such programs due to smoking cessation requirements, lack of transportation, insufficient dedicated resources, or concerns about feeling “worthy” enough.^16^^,^^36–40^ COPD is potentially fatal; yet, interpersonal discomfort on the taboo topic of death may delay patient-provider discussions on prognosis.^41^ The lack of dedicated resources for COPD has also extended into research,^42^ as evidenced by some researchers calling COPD the “poor cousin”^25^ of other chronic conditions.

**Figure 1.**
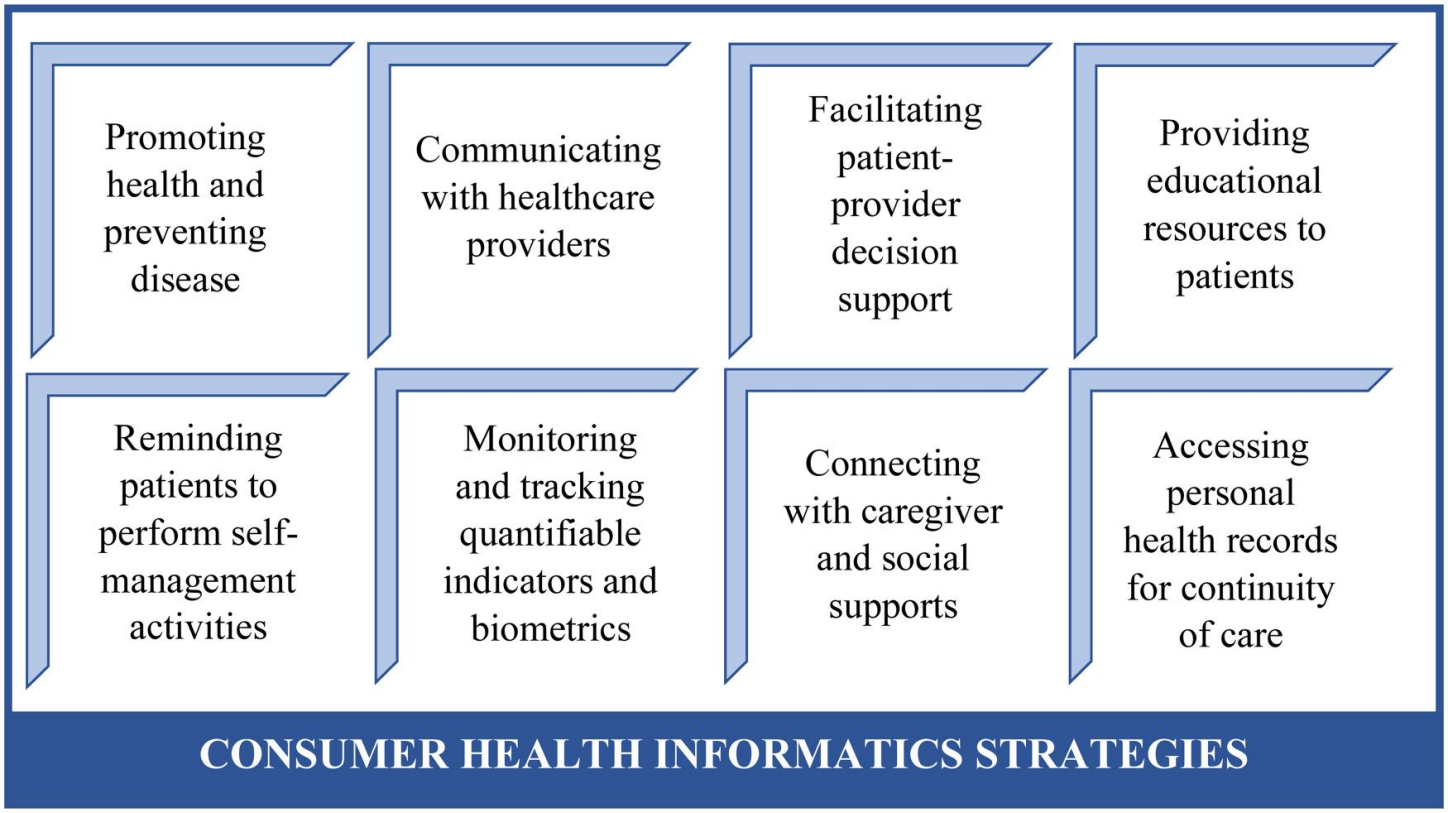
Common consumer health informatics strategies.

Consumer health informatics (CHI) holds potential for addressing challenges related to health outcomes and resource access for people with COPD. Figure 1 illustrates common CHI strategies applied across different health conditions that update and expand upon Klasnja and Pratt^43^ 5 intervention strategies for mobile health. These include health promotion and illness prevention, healthcare provider communication, decision support, patient education, self-management, monitoring and tracking, caregiver support, and continuity of care.^43–49^ To date, most CHI interventions for COPD have involved telehealth, mobile apps, and tracking devices to (1) promote disease self-management through information and education^50–58^ and (2) facilitate the monitoring of physical activity and COPD symptoms.^50^^,^^51^^,^^53^^,^^55^^,^^58–60^ However, the effectiveness of such tools for COPD is uncertain,^51^,^61^ perhaps due to the lack of socioeconomic support required to “self-manage” COPD effectively.^53^^,^^62–64^ COPD apps illustrate the dominance of self-management strategies,^53^^,^^60^^,^^61^^,^^65–68^ while also repeatedly demonstrating the limited evidence of the effectiveness of these apps,^58^^,^^65–68^, and the lack of examples in addressing social needs for self-management.^53^^,^^58^^,^^60^^,^^61^^,^^65^^,^^66^

Many of the CHI strategies for COPD have evolved from interventions for other chronic conditions (eg, diabetes and hypertension),^69–71^ which typically do not have the same clinical and social characteristics as progressive, fatal illnesses with few treatment options like COPD.^68^ In addition, even when internet-based interventions are extending to support emotional and mental health for people with chronic conditions, the focus continues to be on other illnesses, such as cardiovascular disease, diabetes, chronic pain, and cancer.^72^ Moreover, the design of COPD CHI interventions may not incorporate COPD patients’ views.^53^ Consequently, CHI interventions for people with COPD may not be addressing needs regarding social isolation,^5^^,^^9^ countering stigmatization,^12^^,^^15–17^ and supporting mental and emotional health.^20^

This gap is especially significant given that digital technologies (DTs) have the potential to transform stigmatizing, isolating experiences and inequitable outcomes by providing a place for the narration and validation of experience,^73^^,^^74^ peer-based information exchange,^73–75^ emotional support,^73^^,^^74^ and community building to advocate for resources and policy change.^76–78^ We therefore aimed to explore how DTs were used by people with COPD to promote their physical, social, emotional, and mental health.^72^ In this research, we take COPD as an example of a progressive, potentially fatal illness for which clinical and social characteristics may lead to fundamentally different illness experiences than those for which CHI interventions are typically developed.

### Research objective

To investigate how people with COPD use DTs, we sought their perspectives on the current and potential roles of DTs in addressing illness experiences, outcomes, and social connectedness.

## Methods

### Theoretical foundations and definitions

**Figure 2.**
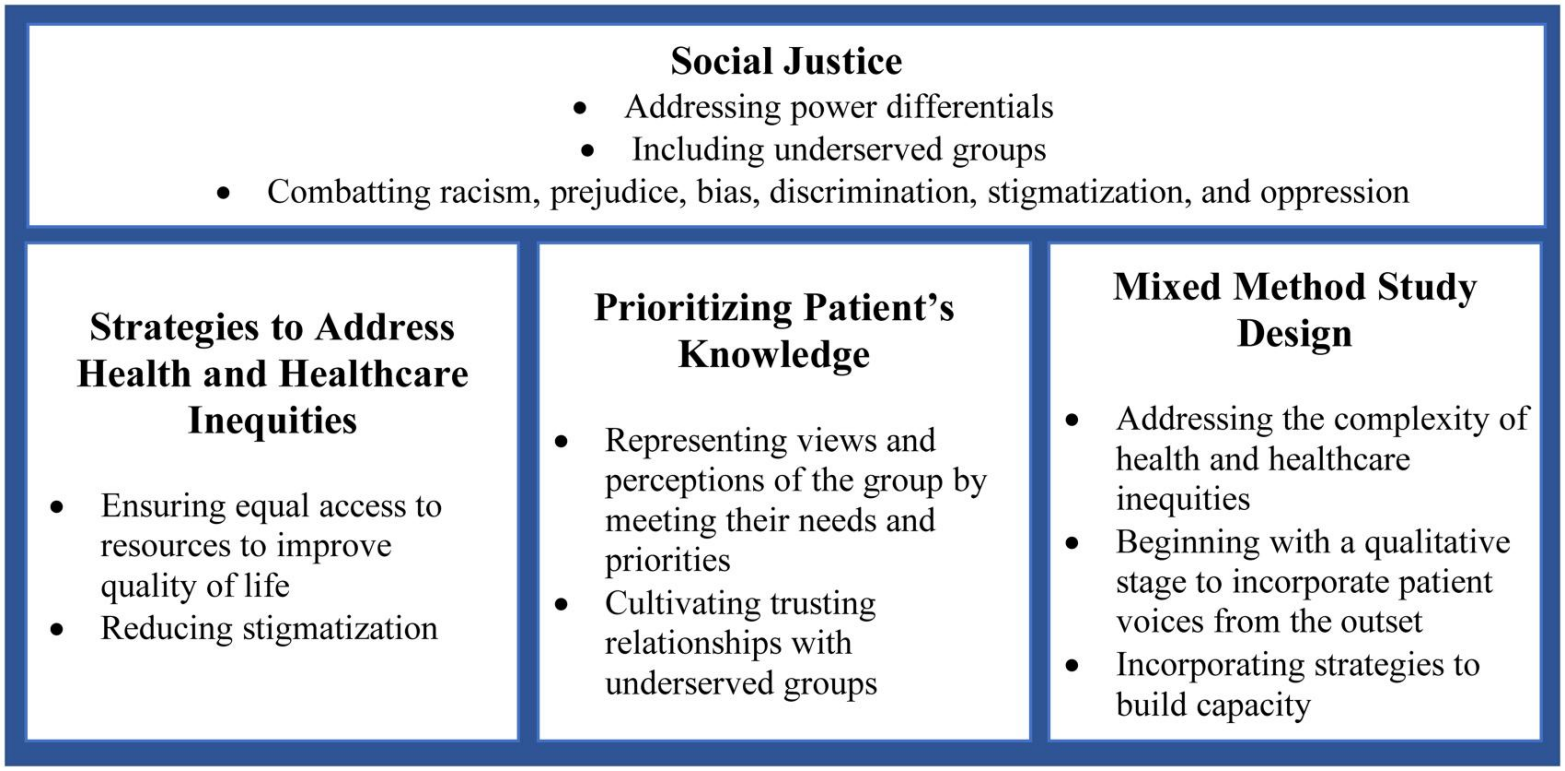
Transformative approach for consumer health informatics.

We applied the transformative approach,^79–88^ rooted in social justice and equity, throughout all study stages. Figure 2 illustrates that the core principles of the transformative approach can be conceptualized to study how CHI may meet patients’ needs and priorities, address power differentials, and combat discrimination and oppression.^79–88^ Our use of the transformative approach prioritizes patient knowledge about access to resources for improving quality of life, reducing stigma, and promoting social inclusion.^79–83^^,^^85^ Transformative mixed method studies share participatory methods with community-based participatory research,^89–91^ and often begin with a qualitative stage to incorporate participants’ perspectives into the research design, with the aim of elevating participants’ knowledge to challenge an inequitable status quo.^83^^,^^85^

Our study asked people with COPD how they use DTs to support their experiences and outcomes with COPD and for social connectedness. To avoid assuming the scope of DTs used by patients, we provided multiple examples of DT activities (eg, emailing, text messaging) and tools (eg, Skype) and designed our questions to consider different potential roles for DT (eg, communication, education, information-seeking).^73^^,^^74^ We defined *social connectedness* as the sense of belonging people have with their family, friends, community, and peers.^92^ Our definition of *illness experiences and outcomes* evolved from definitions of quality of life^93^ and structural inequality^94^^,^^95^: we considered the individual’s perspective of their physical, social, emotional, and mental health and the historical, economic, political, and societal contexts that shape this perspective. Notably, we use the term *fatal illness* to reduce the taboo around discussions of death^41^ and to draw attention to the potential outcome of COPD.^96^ However, we also want to emphasize the importance of matching individual preferences for the terms people living with these illnesses use when describing their illness trajectory.^97^

### Data collection and study design

**Figure 3.**
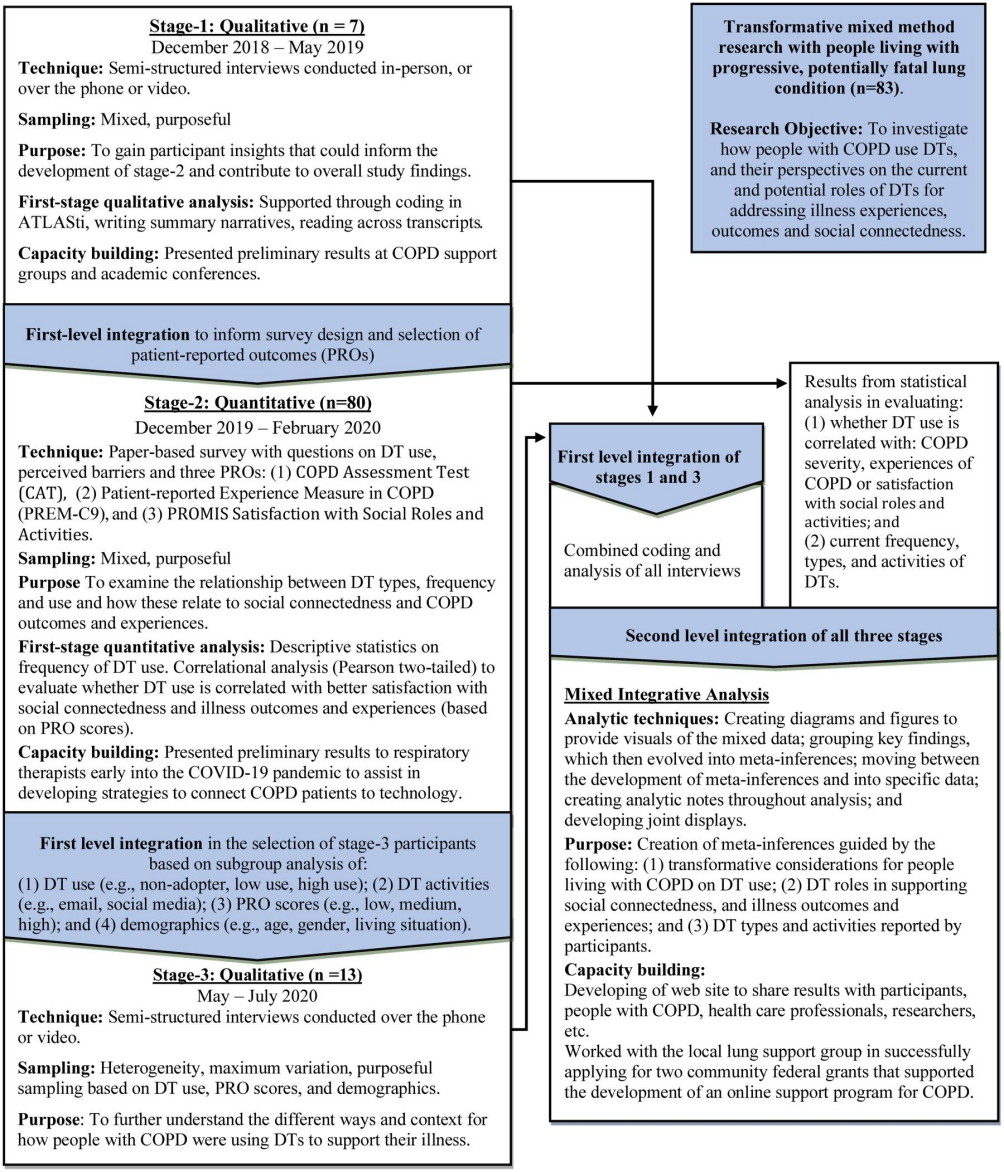
Integration of transformative mixed method study.

A transformative exploratory mixed methods research study^79^^,^^80^^,^^83^^,^^85^^,^^86^ was conducted from December 2019 to July 2020. Figure 3 shows the 3 sequential data collection stages:

Stage-1: Semi-structured interviews conducted in-person or remotely, via video-conferencing or telephone, to inform the selection of measures and overall survey design.

Stage-2: Paper-based survey incorporating questions about DT use and common perceived barriers,^71^^,^^98–100^ and 3 validated patient-reported measures (PROs): the COPD Assessment Test (CAT), Patient-Reported Outcomes Measurement Information System (PROMIS) Satisfaction in Social Roles and Activities, and the COPD Patient-Reported Experience Measure (PREM-C9).^101–104^ The survey was pilot-tested prior to launch with 1 stage-1 participant, and reviewed by academic survey designers.

Stage-3: Semi-structured interviews with survey participants, conducted remotely via video-conferencing or phone.

The PROs were used to evaluate if people who used DTs to support their COPD or for social connectedness would report better PRO scores, to assess the severity and experiences of having a potentially fatal lung condition, and to assist in identifying stage-3 participants. PROs were selected for this study based on their alignment with the transformative approach in having “the status of a patient’s health condition… come[s] directly from the patient, without interpretation of the patient’s response by a clinician or anyone else”.^105^^,^^106^ The National Institutes of Health (NIH) has invested extensively in the development of PROMIS in creating more relevant measures for evaluating quality of life and day-to-day functioning for patients with chronic disease.^107^ Both the NIH and the US Food and Drug Administration have recommended the use of PROs for clinical research.^107^^,^^108^ The CAT^102^ has been extensively use in clinical trials,^109^ clinical practices for evaluating severity of COPD, as a screening measure in addressing the underdiagnosis of COPD,^22^ and in health informatics research in evaluating self-management apps.^60^^,^^65^^,^^66^ Recent Global Initiative for Chronic Obstructive Lung Disease updates to COPD guidelines have recommended CAT be used to address the limited reliability of using FEV1 to evaluate COPD severity at the individual level.^4^

The study received harmonized ethics approval across 5 health regions and 2 universities in British Columbia (UBC REB# H18-01530).

### Participant sampling, recruitment, and inclusion criteria

The 2-dimensional sampling model consisted of multilevel mixed purposeful, convenience, and nested sampling.^110^^,^^111^ This sampling model involved recruiting 6-12 participants for stage-1, 65-70 participants for correlational analysis of stage-2 survey data, and 3-4 participants in subgroups for stage-3 interviews.^111^

Following this sampling model, participants were recruited from community and health organizations across British Columbia, Canada who provided support to older adults and the COPD population. Our initial inclusion criterium was that people had to identify with a diagnosis of COPD. However, early into the study people came forward who had shared lived experiences of a progressive, potentially fatal lung condition, and some were attending the COPD support group when they could find no available support related to their illness. In order to surface this hidden population’s experiences, we broadened our inclusion criteria to people living with a progressive, potentially fatal lung condition and identified with the COPD community. The survey began with 3 questions: (1a) Do you have COPD, emphysema, or chronic bronchitis; (1b) If not, what makes you a good fit for the study?; and (2) Have you been formally diagnosed with COPD. In addition, the results from the PROs were used to further assess inclusion in the study based on people’s severity (CAT) and experiences (PREM-C9) of living with a progressive, potentially fatal lung condition.

### Data analysis

Interviews were audio-recorded and transcribed verbatim, then coded using ATLASti software.^112^ A codebook for both interview stages was created and consisted of concept, versus, attribute, and *in vivo* codes.^113^ Interviews were coded at the end of stage-1; all interviews were merged as a set at the end of stage-3.

Paper-based surveys were entered and analyzed through SPSS. An analysis guide was created with a statistician before conducting Pearson’s correlation testing. Descriptive analysis was conducted to evaluate frequency distribution for types and activities of DTs and to assist in identifying stage-3 participants.

Our integrative mixed-method analytic approach^114^ involved comparing and contrasting data across the 3 stages to develop meta-inferences. As illustrated by the triangular boxes in Figure 3, data were mixed after each stage and after completion of the 3 stages. Our analytic process of integration involved creating figures and joint displays to provide visuals of the mixed data.^114^^,^^115^

## Results

### Characteristics of participants

Participants (*N* = 83) included 3 who completed only the stage-1 interviews (*n* = 7 total). Eighty completed the stage-2 survey. Thirteen survey respondents also completed stage-3 interviews. Multimedia Appendix SA details the demographics and PRO results of all participants. The data presented in the results represent the 77 participants who identified as having COPD. The results from the people who identified with a lung condition outside of COPD are summarized in the final section of the results and supporting data are presented in Multimedia Appendix SC. Of the 77 COPD participants, 3 were unclear about their COPD diagnosis (3/77; 5.2%) due to hearing different messages between providers, lack of follow-up, and recently receiving a lung transplant. COPD participants were close to high severity of COPD (19.38 ± 6.49), with an average age of 74 ± 7.9. Most of the COPD participants were White individuals (83.1%), over two-thirds reported encountering financial difficulties at some point, and 1-quarter had a postsecondary degree. Notably, 32 of the 47 women lived alone, whereas 1 man lived alone.

### General use of digital technologies among people with COPD

Most COPD participants across all 3 stages (67/77; 87.0%) reported use of DTs. People who reported limited or moderate use tended to use 1 or 2 devices, while people categorized as “high use” (36.4%; 28/77) often used 3 or more devices. The main devices used by technology adopters were mobile phones (51/67; 76.1%), desktops (74.6%; 50/67), tablets (67.7%; 44/65), and laptops (61.5%, 40/65). Of those, mobile phones had the lowest percentage of high use (34.3%; 23/67). Notably, over half (33/64; 51.6%) of technology adopters reported that they had not downloaded apps; some participants first discovered they had a health app when they scrolled through their mobile devices during the interview.

### DT use for social connectedness

The survey demonstrated that people who used DTs reported using them most often to connect with their family (53/64; 82.8%) face-to-face friends (54/68; 79.4%), and online friends (46/68; 67.6%) (see Multimedia Appendix SB for a summary of technology use and frequency). Close to half of DT adopters who had used DTs to connect with their health professional (34/67; 50.7%), or other people who supported their care (27/63; 42.9%). In the qualitative data, there were limited examples of people using DTs to connect with COPD supports specifically, however, people reported often using to *DTs for social connectedness*. During the stage-3 interviews, people noted how the connections with their family had changed with the onset of the COVID-19 pandemic when they began to meet online for *virtual family reunions* (see Table 1A). No one had conducted a video healthcare visit.

**Table 1.** Current DT strategies for social connectedness and illness experiences and outcomes.

**A. Use of digital technologies for social connectedness**	
**Quantitative**	
	There was a low, positive correlation between more frequent of DTs for COPD and satisfaction with social roles and responsibilities (PROMIS PRO) (*r* = 0.35, *P* (2-tailed) < .01). There was a low, negative correlation between more frequent use of DTs feeling less isolated by technology, *r* = −0.47; *P* (2-tailed) < .01.
**Qualitative**	
	**Technology for social connectedness** *[Lists groups of connections]… You know the technology comes in handy for lots of connecting. So, yay, it is really amazing what you can do. *[PA06, Stage-1] **Virtual family reunions** *I just started using WhatsApp here maybe, not even two months ago. One of my stepsons set it—well he didn’t set it up, I set it up but he wanted to do it so that was… so we get the whole family on there*. [PA61, Stage-3] *My daughter organized it… I just went down to her condo and talked to mostly family members.* [PA06, Stage-3]
**B. A “lack of a cure” and a “lack of information” on progression and treatment**	
**Quantitative**	
	A low correlation was found between frequency of DTs and experiences of COPD (PREM-C9) (*r* = 0.26, *P* (2-tailed) < .01), and no correlation was found between frequency of DTs for COPD and outcomes of COPD (CAT) and experiences of COPD (PREM-C9). The PRO question which asked people if they had enough information about their condition (0 positive experience) versus being frustrated by the lack of information about their condition (5 negative experience) had a mean of 2.07 ± 1.36, suggesting a need for more information about their condition. The PRO question which asked people if they understand their COPD treatments (0 positive experience) versus felt confused about how their COPD treatments worked (5 negative experience) had a mean of 1.85 ± 1.36, suggesting a need for more information about how their COPD treatments worked.
**Qualitative**	
	**Lack of a cure** *Oh there’s no cure, there’s really no treatment—but nobody says ok, here’s what you’re going to go through, here’s what the progression of it is like.* [PA67, Stage-3] *There’s no like cure or anything but if they can give you an idea of what might relieve symptoms that would be something that [I] would be interested in.* [PA64, Stage-3] **Lack of technologies and information for COPD** *With heart disease there were so many tests done to determine what damage my heart has undergone, but they have imaging, .. blood tests, … angiograms,… hands on testing like listening and… echocardiograms…– so they have a number of diagnostic tools. But with COPD the diagnostic tools… I’m sure they’re very well based in science and experience, but it just seems pretty minimal compared to another organ’s disease and yet COPD or chronic lung disease is such a common problem… There just seems to be little depth to it.* [PA67, Stage-3] *They give you the testing for the amount of air, or oxygen you’re not getting and they in the lungs…so, they can tell you, well, you are getting worse each year and they can't tell you exactly where [you are at]. *[PA61, Stage-3] *It’s progressed. When I first started with Symbicort it was one puff, once a day and now it’s two puffs, twice a day. SO this is how you know [the progression], you just increase dosages of switch inhalers, or go to something a little stronger or longer lasting.* [PA15, Stage-3] *Just recently, it’s taken a turn for the worse and my doctor just recently referred me to the lung transplant place. So that’s kind of a hard one to take and I’ve done a few of the testings they want.* [PA04, Stage-1] *[Starts reading doctor’s notes]. She’s got “very severe COPD”. First time I knew I was very severe….So I’m like “Okay, so what does this mean?” He says “well, technically uhm, as you age, you’re going to lose a couple of points every year”. He didn’t say what the level of my points were, whatever. So I went home and read all about it and everything. And then I got a new doctor, and same thing! Just don’t pay any attention. Like, oh, I see you have COPD—and, they keep sending me off for tests.* [PA11, Stage-3] **Lack of information on recent medication and treatment advancements** *So far all I’ve been able to discover about COPD in terms of treatment is puffers, that seems to be it. And that can’t be right. I mean to me that just doesn’t make sense, there must be other treatment right? Now is that wishful thinking, I don’t know. I know humans get things that are untreatable*. [PA67, Stage-3] *If they start when they’re younger being taught how to take their medication properly, and more importantly to understand … have a doctor really understand and be able to tell you why you have this condition and what the reason is and what the outlook will be, you know, if you take this medication.* [PA78, Stage-3] *I think it is [hard] to find out really, you know, whether you have to be on the medication, you know, and really how bad their lungs are.* [PA04, Stage-1] **Follow-up and the need to advocate** *I am surprised that there has been absolutely NO follow-up on the original diagnosis made 15+ years ago. I have no idea if my condition is better or worse!!* [PA 14, open text survey response] *Filling in the blanks of lack of information shared by doctors and people that should know… research, and advocating for myself when I decide there's a need for more information*. [PA04, Stage-1]
**C. Strategies for self-management**	
**Quantitative**	
	The mean for the PRO question that if participants were confident (0) versus worried (5) that in a flare-up they would have access to treatments or a doctor/nurse was 2.00 ± 1.46, suggesting that people were somewhat worried about being able to access treatments. The mean for the PRO question that asked participants if they were not worried (0) versus worried (5) about getting care from health professionals during a flare-up was 1.95 ± 1.43 suggesting that people were somewhat worried about being able to access care from a health professional during a flare-up.
**Qualitative**	
	**Impacted by the lack of information** *I started a program of constantly breathing through my nose and out through my mouth and pushing the air out. If I do that more every day is that going to help? I don’t know. I can only go from my experience and try to live with it.* [P78, Stage-3] *About a year ago I was starting to notice that I was coughing excessively… I was online reading… and it looked to me like I was starting to pick up some of the side effects… So I stopped using that [medication] and the cough kind of subsided and went away, although I still cough to bring up mucus and stuff, [p]…. And now I don’t have anything and that’s kind of what triggered me to maybe go back and maybe get revaluated, to see if I can maybe get something else*. [PA61, Stage-3] **Action plan** *At my annual checkup he allots about 40 minutes, and we do what he calls the “big talk”, we chat, you know, how are you doing, blah, blah, blah. And he’s developed an action plan, you know, for me as well and printed that out for me too and you know, so this is where we’re going, if you get sudden panic attacks and stuff, and this is how you deal with it, things like that. So no, he’s been really, really supportive*. [PA11, Stage-3] **Importance of being on a long-term maintenance plan** *Get into some kind of a program, where there’s experts that could help you with your COPD and your breathing and teach you how to maintain your breathing… It’s just one of the things you have to live with. In the beginning, I was pretty freaked out about it because I didn't understand it. So [now] I pace myself. And that’s what [respiratory therapist’s name] teaches us, just to maintain and take care of ourselves right…. When I first got it, I didn’t pay attention and got quite sick. But now if I, if I start feeling, then I do something about it right away. You know I don’t sit at home going, oh I can take care of this myself.* [PA03, Stage-1] *I’m in kind of a maintenance portion of that, so I go twice a week to the hospital. So do you find out a little there, not so much now that I’m in maintenance program. When I was going three days a week, one day they had an education component to the program, and so I would learn through our leader, she’s a respiratory therapist that runs the program at the hospital. Yay, so that’s how I was able to do that.* [PA02, Stage-1]

People who reported more frequent use of DTs for social connectedness reported higher satisfaction with their social roles and activities. Non-adopters were more likely to report that technology isolated them, but 2 high adopters of technology also noted that technology compounded their feelings of isolation in being unable to connect to resources, friends, and family.

### Current DT strategies for COPD experiences and outcomes

Participants frequently used the phrase “lack of*”* regarding information about their diagnosis and about progression and treatments. For participants, the role of DTs in illness outcomes and experiences was limited in part due to a “lack of*”* available resources. Additionally, participants felt that the “lack of*”* information on prognosis and treatment corroborated their status as members of an underserved illness group. Furthermore, since few DTs directly addressed their illness experiences, they expressed a “lack of*”* interest in using current DT options for COPD, though some did use existing DTs in line with current CHI strategies.

#### A “lack of” cure and a “lack of” information about progression and treatment

Participants referenced the “*lack of*” information not only in terms of diagnosis; they also associated the *lack of a cure* with the lack of information about progression and treatments for COPD (see Table 1B). The *lack of technologies and information for COPD* resulted in participants evaluating *their illness progression* through changes to their treatments. Many participants wanted more tailored information about their current stage of COPD and their illness’s progress. They also wanted to know about *updated medication and treatment advancements* that had been prescribed by healthcare providers to some people within their COPD group, and whether their current, long-term medication was still being effective. Some participants noted that they had received no *follow-up* information from their healthcare providers since their diagnosis, and others noted having to *advocate* for themselves when they needed more information about newer, approved therapies for COPD from their healthcare provider.

#### Strategies for illness self-management

Similarly, strategies for illness self-management were *impacted by thelack of information* on COPD management, medication, and prognosis (see Table 1C). People who lacked access to a provider offered examples on how they were managing their illness by discontinuing medications that were perceived to no longer be working, or developing breathing exercises while being uncertain if they were helpful. Other people cited their *action plan* as the primary resource received from their healthcare provider and knew when to reach out for services. However, these management strategies were quite different from those of the 2 participants with access to an ongoing pulmonary rehabilitation program; both noted the *importance of their multi-yearmaintenance programs* for providing them with regular connections and tailored information for managing their COPD.

#### Interest in current consumer health informatics strategies

*Information seeking*: Potentially linked to the perceived “lack of” information from healthcare providers, COPD patients’ most common DT uses involved searching for information (see Multimedia Appendix SB). Participants were more likely to seek information on symptoms and medications and treatments, and some found it could be difficult to assess the accuracy of online information or be “mentally upsetting” to read statistics about their illness (see Table 2A).

**Table 2.** Interest in using current consumer health informatics strategies.

**A. Information seeking about COPD**	
**Quantitative**	
	Participants reported searching online for information on: **Symptoms** (non-use 12/67, 17.9%; limited use: 34/67, 50.7%; high use: 21/67, 31.3%) **Medications and treatments** (non-use 13/67, 19.4%; limited use: 38/67, 56.7%; high use: 16/67, 23.9%) **Statistics of COPD** (non-use 27/68, 39.7%; limited use: 30/68, 44.1%; high use: 11/68, 16.2%)
**Qualitative**	
	**“Lack of” Information** *I did, I did [go online] in the beginning, but not really, not really much now, ‘cause I know what happens to COPD, like you know they say the dos and the don’ts.* [PA03, Stage-1] *I’ve got really enough printed information that I got through my doctor and my pharmacist and COPD support group were really helpful….I mean so you’re gonna do the research and access it once or twice and by that point you’ve pretty much got all the information you need, you know, so [pause] you know, why keep delving into it every day*. [PA15, Stage-3] *You can look at a bunch of different sites and compare the information but I have no way of knowing which site is more accurate than another… like fake news. But I think in the end, the doctor is the one that’s gonna give you the, hopefully, the better information and the more real information.* [PA64, Stage-3] *[There's the need] to dig in and search because mentally it can be quite upsetting to know that you have a terminal disease, right? And that you don’t know what’s going to happen in the future.* [PA02, Stage-1]
**B. Monitoring and tracking for COPD**	
**Quantitative**	
	Participants who used DTs reported the following in using DTs for tracking and monitoring: **Other health measures** (eg, heart rate, blood pressure) (non-use: 27/66, 40.9%; limited use: 18/66, 27.3%; high use: 21/66, 31.8%). **Oxygen levels** (non-use 43/65, 66.2%; limited use: 10/65, 15.4%; high use: 12/65, 18.5%); **Daily activities** (eg, daily steps) (non-use 43/63, 68.3%; limited use: 9/63, 14.3%; high use: 11/63, 17.5%); **Medications** (non-use 39/66, 59.1%; limited use: 15/66, 22.7%; high use: 12/66, 18.2%).
**Qualitative**	
	**Oximeters** *I take my blood pressure every now and then because that’s something you can't tell. But with my breathing I know when it’s problematic and when it’s not.* [PA15, Stage-3] *I was dead! for fifteen minutes! [pause]My doctor was basically explaining that the C0_2_ can build up when you're breathing shallow… so now I know if I get a good oxygen level [on my oximeter], but I'm out of breath, I have to do the extra job of extra inhale exhalation. And this [points to oximeter] has been my savior.* [PA05, Stage-1] *I was checking [my oxygen level] when I wasn't doing well and it would go down but come back up when I stopped and did my breathing exercises so I don’t think I’ll ever be on oxygen because it doesn’t go… my oxygen level doesn’t go low enough.* [PA41, Stage-3] *About three weeks ago my reading was 100! So, I know! It’s like I've got all kinds of my friends, we’ll be sitting around, I’m like “oh here, check your blood [oxygen]”. And it’s sort of like a party favorite. Even the guy at the gym did it. I'm like “oh let me see”. And I’m like, oh you're only at 97*. [PA11, Stage-3] **Monitoring and tracking for a progressive, potentially fatal illness** *As far as myself tracking it, uhm, I don’t really have anything. Nothing concrete. Just what I can remember in my head.* [PA04, Stage-1] *There’s one here that says welcome to the new health app. But I haven’t got it set up… I don’t know what it could do for me, if it could do anything… [My girlfriend] bought an Apple one and she gave me this one. I put it on and I was using it for a while. It tells you how many steps you walked that day and I just don’t wear it anymore.* [PA44, Stage-3]
**C.** **Asynchronous versus synchronous support**	
**Quantitative**	
	Participants who used DTs reported the following frequencies in: **Reading posts on social media sites, blogs, or online forums** (non-use: 29/66, 43.9%; limited use: 19/66; 28.8%; high use: 18/66, 27.3%) **Commenting on social media sites, blogs, or online forums** (non-use: 50/67, 74.6%; limited use: 15/67; 22.4%; high use: 2/67; 3.0%)
**Qualitative**	
	**Synchronous** *You see who’s online when you type in messenger [you can see] who’s awake, yay your just click on them and away you go. I do that often.* [PA-03, Stage-1] *Well I think [a video conference with a healthcare provider] it’s the only way to go. And again it’s always nice to put a face to somebody that you’re talking to. It makes a big difference.* [PA44, Stage-3] **Asynchronous** *But most of Facebook is telling you what they had for breakfast, and you, and I’m not interested in having conversations like that.* [PA06, Stage-1]. *Well some people keep it more and some people text or make comments, it’s just not something I do. I’m interested in reading what they’re doing.* [PA41, Stage-3] *I’ve been having concerns about my COPD so because you don’t get to see your doctor anymore I’ve thought, well maybe I should look online and then I kind of lose the motivation to do it for some reason. I don’t know why. It’s such a one-way communication system*. [PA67, Stage-3]

*Monitoring and tracking:* The majority of participants reported no (39/66; 59.1%) or limited (15/66; 22.7%) use of DTs for tracking medications (see Table 2B). Interviewees stated that bubble packs for their medications met this need, so DTs weren’t necessary. Although pedometers and oximeters have been promoted for COPD—for tracking physical activity and monitoring oxygen levels, respectively—two-thirds of people did not use devices for these purposes. Yet 59.1% of participants reported using DTs to *monitor and track* other health conditions (eg, hypertension). Interviewees who had previously used oximeters found that oxygen percentage information was of limited value in early-stage COPD, as their oxygen readings did not reach critical levels. In addition, people knew when their breathing was “problematic and when it’s not” without technology*.* However, a participant in late-stage COPD found the oximeter to be a “savior“ for determining whether their shortness of breath was related to their O_2_ or CO_2_ levels.

*Asynchronous versus synchronous support:* Although the majority of technology adopters reported reading forum or blog posts, only 2 people reported high frequency in commenting on forums, blogs, or social media (see Table 2C). Eighteen survey respondents reported using DTs frequently to participate in support programs. However, the open-ended survey and interviews revealed participants most often used phone and email to communicate with their peer support group. When reaching out to people to discuss their illness preference was for *synchronous* forms of communication, rather than public, *asynchronous* forms of communication, such as Facebook.

### Use of DTs to support quality of life among people with COPD

During interviews, participants offered few examples of using DTs to manage their COPD specifically. However, they noted an interest in connecting with people with shared illness experiences, and many steered the conversation toward explaining their use of DTs to support their social, emotional, and mental health beyond COPD symptom management.

#### Creating a safe online place to meet

People who were interviewed after the start of the COVID-19 pandemic mentioned the *loss of their connections* with peer support groups and expressed interest in creating a *safe online place to meet* (see Table 3A). The in-person COPD group had been a valuable place for people to *share lived experience*s: to find information on treatments that worked for others, for discussions about the *fatal* aspect of COPD, and to know that they were not alone. However, participants also noted that having these existential discussions necessitated first knowing the other people and that 2-way communication was important.

**Table 3.** People with COPD’s use of DTs in supporting quality of life.

**A. Creating a safe online place to meet**	
**Quantitative**	
	Few people reported using DTs to participate in: **Group text chats** (non-use: 55/67, 82.1%; limited use: 8/67, 11.9%; high use: 4/67; 6.0%); or **Video calls** (non-use: 47/67, 70.1%; limited use: 11/67, 16.4%; high use: 9/67, 13.4%).^a^
**Qualitative**	
	**Loss of their connections** *It’s made everything difficult because this COVID is making it that much more isolated. I was isolated before COVID come along and you would think it would make that easier but security is tighter.* [PA32, Stage-3] *The major thing is that I don’t get to go to [my COPD group] anymore, all of those kinds of support groups are cancelled of course. Although it only meets once a month or so it is a little bit of extra support and education, and you pick up a little bit of knowledge here and there.* [PA67, Stage-3] **Safe online place to meet** *I would love to meet somebody with a little bit more functionality than me. But you’re afraid to go on web, on dating sites, and things that because you don’t want to be confessing COPD, or you know, and again, it’s unsafe. Just a community of people that you can talk to so that you find people and connect with them. I mean that’s how you make friends*. [PA11, Stage-3] *What it would look like for me would be an opportunity to have conversations like I’m having with you right now. Where I could express my concerns, and then once I get past that, trying to find solutions to those concerns with other people. …. And mutual support, what are others like me doing and does it work for them?* [PA67, Stage-3] **Share lived experiences** *Or where somebody, maybe along the lines of throw out a topic. So what’s been your experience with Annora as an inhaler? What did you like about it? What did you not like about it? Ah, it’s simple things like that. Those are the things that, that strike home in our group.* [PA11, Stage-3] *I do ask questions of other people there with COPD if they’ve experienced the kind of things that I’ve experienced. But more often, it’s that does this medication have this side effect… having died once, you are aware that you are.. in a precarious situation, position.* [PA05, Stage-1] *The one thing that we all know that none of us are gonna escape… perhaps there is a wee bit of fatalism in our group. But I guess it’s a whole lot easier, even with this sorrow, to know that we have other people that we could care about rather than go through it alone.* [PA32, Stage-3] *Yea I’m not sure that I’d be prepared to have the type of discussion that you and I are having…with a large group online. I’m a little bit more of a private person than exposing… to the world at large when I don’t know who these people are. But yea, I’ve got no problem discussing with you and with my friends and neighbours, we talk about it*. [PA15, Stage-3]
**B. Intergenerational connections**	
**Quantitative**	
	Participants who used DTs reported the following frequencies in: **Accessing online ancestry sites** (non-use: 43/69, 62.3%; limited use: 16/69, 23.2%; high use: 10/69; 14.5%); and **Writing about life experiences** (non-use: 51/68, 75.0%; limited use: 13/68, 19.1%; high use: 4/68; 5.9%).
**Qualitative**	
	**Connections to grandchildren** *Seeing video of grand child who was born during COVID… But yea, she’s always on there… well with the grandkids and what they’re doing all the time so we got to follow that.* [PA61, Stage-3] *I know, it’s beautiful and her birthday was on May 1st, and she showed me a picture of my great-grandson speaking to her on her phone and there’s a picture of him speaking to her, and wishing her a happy birthday and I thought you know, technology is so wonderful. It really is. It really is.* [PA78, Stage-3] *I’m with Facebook enough where I get to see pictures of my great grandchildren, so that is good.* [PA06, Stage-1] **Carrying on tradition, leaving a legacy, and passing on memories** *My grandmother taught me how to play solitaire when I was just a little kid and I’ve just kind of, you know, I’ve played it with cards over the years but I can play it online, [laughs] I just… it’s something to do. My whole family does the same thing with solitaire, so I don’t feel so alone.* [PA64, Stage-3] *I do use technology to save documents and materials from the past. Like I have much, much material from my father’s life, my mother’s life, my grandparent’s life. So I’ve recorded all of that online so… So mine is open to the public so people can go on there and see historical documents from—actually back to the late 1700’s and early 1800’s of my family.* [PA67, Stage-3]
**C. Leisure activities**	
**Quantitative**	
	Participants who used DTs reported the following frequencies in: **Watching online** videos (eg, YouTube): (non-use: 26/68, 38.2%; limited use: 28/68, 41.2%; high use: 14/68; 20.6%) **Listening to audio recordings or podcasts**: (non-use: 41/67, 61.2%; limited use: 18/65, 27.7%; high use: 8/67; 11.9%) **Playing video games online**: (non-use: 40/69, 58.0%; limited use: 15/69, 21.7%; high use: 14/69; 20.3%)
**Qualitative**	
	**Connecting socially** *I play pool against people from all over the wooooorld [stresses the word]… I have a person who has befriended me and we have become friends on Facebook… I have two lady friends who live in Nairobi and another lady lives in Greece and another fellow lives in Israel, one in France, England, uhm—the United Kingdom, I know people that play there from the United Kingdom, all over Canada*. [PA04, Stage-1] *Yea, I play [games online] with other people. That’s important to me to be playing with other people. Robots are just too boring.* [PA44, Stage-3] **Respite, reminiscing, distraction, and spontaneity** *It’s been a really special thing, reminiscing. I belong to my old high school alumni…. That kind of thing it’s really cool.* [PA67, Stage-3] *We watch a lot of wilderness programs that are on You-Tube. I love You-tube…Yea I love nature, I love outdoors. I spend a lot of time outdoors. I used to be quite an avid hiker and I’m still kind of hunting fish.* [PA61, Stage-3] *I have an app I’m addicted to. I put in stand-up comic. I put the headset on and I get into bed and I will fall asleep, but who cares. But I will wake up laughing… And I think that is one of the best thing I do for myself… Some days I don’t like what’s in my head, but listening to these people and the things that they tell you, you get a whole different slant on life*. [PA11, Stage-3] *[Streaming TV shows] gives you something else to do. It’s not great sitting all the time, but it is… and you learn a little something here and there. Yeah, it’s sort of like when I watch Netflix, it’s sort of like having company, you know? So I find it positive.* [PA41, Stage-3] *Mostly what I watch and do almost on a daily basis is tune into Celtic Thunder and listening to their singing and they have so much fun together. And then sometimes I’m lucky enough for whatever happens, it switches over to something entirely different. This one evening it switched over to Johnny Cash and three others.* [PA06, Stage-3] *Cause it takes you down a rabbit hole. You push one web site and it takes you to three others.* [PA11, Stage-3]

a Notably, this was reported 3 months prior to the COVID-19 pandemic.

#### Intergenerational connections

When asked about DT use, participants often referenced *intergenerational connections* (see Table 3B). Specifically, they used Facebook, Instagram, YouTube and Skype to stay updated about their *grandchildren’s* lives. These examples illustrated how DTs have enabled people to feel connected across multiple generations: by *carrying on traditions* introduced by grandparents, by *leaving a legacy* for younger family members through posting photos and documents on ancestry sites, and to *pass on memories* by writing about their life experiences.

#### Leisure activities

Participants used online leisure activities for *connecting socially* and for *respite*, *reminiscing*, *distraction*, and *spontaneity* (see Table 3C). DTs allowed people to make connections all over the world, to document past travels and to revisit hobbies and passions. Other participants shared how online leisure activities, such as listening to music and comedy acts or watching movies and concerts, offered distraction from negative thoughts, provided a broader sense of connection, and afforded opportunities for spontaneity.

### Shared experiences of living with a progressive, potentially fatal lung condition

Multimedia Appendix SA compares the survey data from the participants who identified with a fatal lung condition that wasn’t COPD, and those with COPD. Notably, the non-COPD participants reported more severity of their lung condition and less satisfaction in their social roles and activities than the COPD group. As illustrated in Multimedia Appendix SC, all 3 interviewed participants shared similar experiences to the COPD population when recognizing the fatal aspect of their illness, the *lack of information about treatments* and the need to advocate. Their use of DTs to support their illness were also similar to COPD participants: *self-management strategies* did not extend beyond *information seeking*, and the *intergenerational connections* afforded through DTs were important for staying socially connected.

Notably, 2 interviewed non-COPD participants shared their *experiences in being identified with COPD* when they were out in the community or when struggling to find a diagnosis. The third interviewed participant saw himself as an “oddball” in not fitting into any current programs and joined the local COPD support group. He discussed how technology was *magnifying the isolation* he felt in not having available resources specific to his illness.

## Discussion

Although the majority of participants reported using DTs, there were few instances of DTs being used for COPD beyond asynchronous searching for educational resources. This low utilization of DTs for COPD suggests that CHI’s current strategic foci may not align with the needs of the COPD population. We propose 3 reasons for this lack of alignment. Firstly, CHI’s strategic foci on health promotion, illness prevention, and tracking and monitoring may not be ideal for addressing the existential distress and suffering experienced in living with a progressive, potentially fatal illness.^116^^,^^117^ Secondly, current CHI strategies that easily lend themselves to the “quantifiable self” may not align with progressive illnesses that have a less predictable trajectory.^118^ Thirdly, the individualistic focus on self-management may not recognize the required socioeconomic supports,^62^ and may perpetuate blame on patient populations when they fail to adopt DTs that do not meet their needs.^100^

### Quality of life informatics framework

**Figure 4.**
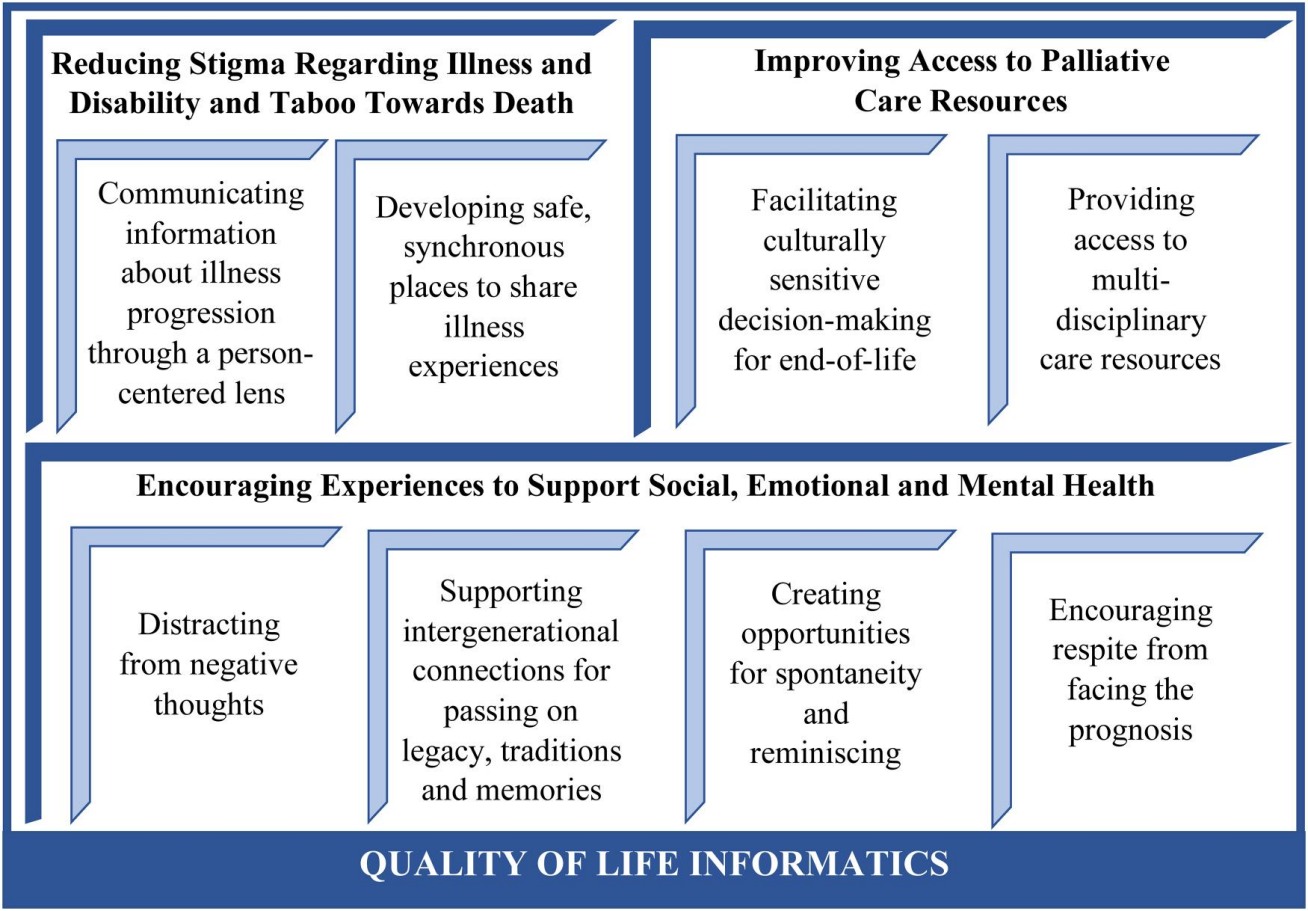
A reconceptualization of consumer health informatics for progressive, potentially fatal illnesses.

Therefore, based on study findings we propose a framework that reconceptualizes CHI toward developing and deploying technologies that center *quality of life.* The proposed framework in Figure 4 provides an alternative to the common CHI strategies presented in Figure 1, and prioritizes (1) reducing stigma,^76^^,^^119^ (2) promoting a palliative approach,^6^^,^^41^^,^^96^^,^^120^ and (3) supporting social, emotional, and mental health.^5^^,^^9^^,^^20^ Exploration of this quality of life informatics framework with study findings is provided in the following sections, and further examples of possible informatics strategies are presented in Table 4.

**Table 4.** Examples of quality of life informatics strategies.

Reducing stigma regarding illness and disability and taboo toward death	
**Framework concepts**	**Possible strategies**
Developing safe, synchronous places to share illness experiences	Virtual community-based programs that brings together patients, families and healthcare providers who can work toward destigmatization and advocate for change.^119^^,^^121^ Internet-based delivery of therapy and pulmonary rehabilitation programs to prevent stigmatizing experiences that may occur when entering public venues for mental health.^72^
Communicating information about illness progression through a person-centered lens	Online training for providers on how to approach culturally-sensitive discussions on illness progression.^97^^,^^122^ Pre-set reminders within electronic health records that prompt providers to evaluate and discuss disease progression with patients annually.^4^ Routine collection of illness-specific PROs (eg, CAT) using patient portals or tablets given to patients to complete as they wait a healthcare visit.^4^
**Improving access to resources for palliative care**	
**Framework concepts**	**Possible strategies**
Facilitating culturally sensitive end-of-life discussions Providing access to multi-disciplinary care resources	Online multimedia that encourages interactive dialogue, self-reflection, and story-telling to support patients and families as the illness progresses.^122^ Patient portals that incorporate tailored resources on advanced care planning^123^ that are more inclusive of patients and families’ cultural and spiritual background,^122^ and incorporate quality of life considerations.^124^ Shared decision-support tools to understand patients’ values and preferences on treatment decisions during their day-to-day activities,^125^^,^^126^ and as their illness progresses. Online decision aids for advanced care planning that incorporate preferences for leaving a digital legacy.^127^ Clinical support tools that use PRO data to recommend referrals to palliative care and mental health resources.^96^ Online synchronous delivery of pulmonary rehabilitation programs with a multidisciplinary palliative care team^128^ to facilitate discussions on living with a progressive, potentially fatal illness.
**Encourage experiences to support social, emotional, and mental health**	
**Framework concepts**	**Possible strategies**
Supporting intergenerational connections for passing on legacy, traditions, and memories	Digital storytelling tools to support passing a on legacy.^129–131^
Distancing from negative thoughts Creating opportunities for spontaneity and reminiscing	Self-care tools that provide recommendations on how to incorporate mindfulness during activities of daily living,^118^ and encourage “digital play”^132^ by matching people to online leisure activities that offer distraction. Internet-based therapy using various modalities, especially cognitive-based therapy (CBT) to support mental and emotional health.^72^^,^^133^ Virtual reality systems for revisiting past travel locations and events^134^^,^^135^ in encouraging spontaneity and reminiscing.
Encouraging respite from facing the prognosis	Recommendation systems that allow people with progressive, potentially fatal conditions to see information about prognosis according to their preferences.

#### Reducing stigma regarding illness, disability, and taboo towards death

Although people mentioned visiting reputable sites for health information, people found the information was not specific enough to their current stage of COPD, and their own personal situation. However, participants expressed the desire for more interactions with their healthcare providers, and some noted how it had been over a decade without follow-up care. Other work has shown that healthcare providers may be reluctant to discuss the progressive, potentially fatal aspect of COPD.^6^^,^^41^ As such our framework emphasizes the need for *reducing stigma regarding illness, disability, and taboo toward death* by encouraging opportunities for *communicating information about illness progression through a person-centered lens*. Importantly, the person-centered^97^ component of this strategy recognizes varying cultural values in discussing illness, disability, and death. As such, these informatics tools must be accompanied by provider training on eliciting patients’ preferences for how and when to approach these sensitive conversations.^97^

People in this study were not interested in participating in the kind of public, asynchronous forums that have been successful with other patient groups (such as those with cancer and HIV^136–138^ when advocating for policy change, addressing stigma, and improving resources and treatment.^139^^,^^140^ Rather, our findings suggested that *developing safe, synchronous places to share illness experiences* could be an alternative way to bring together a community that could work towards reducing stigma. As part of the transformative approach goal of building capacity, the researchers worked with the community partner in developing a virtual program that included biweekly virtual exercise classes facilitated by a kinesiologist and monthly webinars on recent medication and treatment advancements and advanced care planning that were delivered by respirologists, pharmacists and respiratory therapists.^141^ Future research should evaluate whether similar virtual programs could (1) reduce stigma by bringing together a community of patients, families, community members, and healthcare providers that can advocate for change; (2) address the lack of information by providing opportunities to learn from healthcare experts; and (3) support social, physical, and mental health through biweekly peer support.^119^

#### Improving access to resources for palliative care

Chronic care models emphasize that self-management is only 1 piece of care, and the importance of ongoing engagement and follow-up with a multidisciplinary, proactive care team trained on consulting patients on the progression of their chronic illness.^55^^,^^96^^,^^142–144^ However, similar to findings in previous studies,^35–37^ few people in this study had ongoing access to multidisciplinary programs that may help in decreasing depression and anxiety and improving quality of life.^34^^,^^35^. As such, our framework emphasizes the need for *facilitating culturally sensitive end-of-life discussions* and *providing access to multidisciplinary care resources*. Pulmonary rehab programs have been adapted to incorporate leisure activities as a way to target social isolation^145^ and delivered by palliative multidisciplinary care teams who are trained in providing support to people throughout all stages of COPD progression.^128^ The proliferation of teleconferencing tools during the past 3 years^37^^,^^146^ offers a way to reach patients who previously were denied access—those who lack transportation access, have limited social support, currently smoke, or fear viral infection.^37–40^^,^^147^

#### Encouraging experiences to support social, emotional, and mental health

In interviews, many people recognized that “COPD will kill you” and sought support for their social, emotional, and mental health as they faced this reality. As such, they found that CHI’s strategy of monitoring and tracking clinical indicators did not fully align with this need. In addition, our study highlighted that patients may not always follow the treatment plan outlined by providers. As such, there is a need for developing “mundane technologies” that align with the activities of day-to-day living and reveal how patients make decisions around treatments.^125^^,^^126^ However, previous research has noted how monitoring and tracking can negatively impact patients’ quality of life^148^ by being overwhelming,^117^ incompatible with daily life,^149^ and in opposition to patients’ need to maintain control and establish normalcy.^150^ Moreover, medicalized devices that provide continuous monitoring may counter the needed distraction that may be beneficial for reducing negative moods,^151^ and detract from DTs’ role in *distancing from negative thoughts*^152^^,^^153^ when facing the uncertainty of the disease trajectory. Below we examine how participants use of DTs for intergenerational connections, leisure, and other activities outside of their illness provides multiple avenues for distancing from negative thoughts.

Participants’ *intergenerational activities of leaving a legacy, carrying on traditions and passing on memories* could also be a way to prepare for end-of-life, promote well-being, and reduce anxiety for older adults.^154^ Previous studies of people with a fatal illness have noted the importance of “needing to prepare”^6^ by writing wills and advanced planning guides,^155^ and putting legacy documents^116^ in order. Recent research studies have explored how DTs can support leaving a legacy^153^ through the sharing of stories through text, images, videos, and music.^130^ Older adults’ “digital storytelling” can promote emotional and mental health, address loneliness,^131^^,^^156^ and help develop meaningful social connections across generations.^129^ However, although a “digital legacy” can be of comfort to loved ones, without clarity as to the disposition of this information after a person’s death may go against what they envisioned.^157^^,^^158^ Future CHI studies could examine both how digital storytelling and digital legacy discussions could be integrated into palliative care programs.^157^

The online leisure activities participants shared in watching concerts, listening to comedy acts and music, and revisiting past hobbies demonstrated how *creating opportunities for spontaneity and reminiscing* can support social, emotional, and mental health.^159^^,^^160^ Leisure activities can be a venue for countering sorrow from the diminished opportunities for spontaneity and social connectedness among people aging with a chronic illness.^142^^,^^145^^,^^161–164^ Spontaneity has been recognized as promoting social and emotional health for children,^165^ but we were unable to find similar studies for adults. Future research could examine if DTs can be used to prompt spontaneity for older adults living with chronic conditions.

Older adults participating in *active* leisure activities that have been historically pursued in-person (eg, cultural events, volunteer work, hobbies) have been found to be more important in maintaining social connectedness than *passive* activities (eg, using computers, watching TV, or listening to the radio).^166^ However, given participants’ online leisure activities and the evolution of DTs since 2020,^167^ further CHI studies about how both active and passive online leisure activities may support social, emotional, and mental health for people with progressive, potentially fatal illnesses are warranted. For example, CHI studies could expand on emerging research on virtual reality that supports travel-based activities and opportunities to^134^ promote social and emotional health for older adults.^134^^,^^135^

Participants’ limited use of DTs suggest that people with COPD may wish to create a separation between their illness world and their online world. When living with the reality of a potentially fatal illness, people may want choose when to acknowledge their COPD through DT use and when to retreat as a form of *respite from facing the prognosis*. Paterson’s^161^ model of shifting perspectives of chronic illness further illustrates how patients want to maintain control over when to focus on living well and versus the burden, progression, and stigma of their illness. The model illuminates how algorithms may deny people this control, by using previous searches to send unwanted online recommendations during needed respite periods. Thus, future CHI research should evaluate the impact of recommendation systems on the emotional and mental health of people with fatal, stigmatizing chronic conditions.

### Limitations

Heterogeneity could not be achieved in some aspects of the study. Most people identified with a European descent. Most participants attended a COPD support group, so they may represent a more socially connected population. Additionally, not everyone in the study was living with a formal COPD diagnosis. Our analysis of this subgroup illustrated how they can experience another layer of social isolation from not being able to find resources specific to their illness, although some of these participants were partially able to address this isolation by connecting with a COPD peer support group where they could share similar experiences. Other than the PROs, the questions in the survey did not undergo formal validity testing. To address this limitation, existing surveys were reviewed, and multiple people evaluated the survey before it launched.

## Conclusion

Participants’ emphasis on the lack of information about progression and treatments demonstrated the ongoing gap in providing dedicated resources for people with COPD. In addition, the limited examples of DT usage for COPD suggest that current CHI strategies may not meet the needs of the COPD population, and possibly others with progressive, potentially fatal conditions. Significantly, however, the ways in which people with COPD used DTs provides an alternative approach for CHI to develop interventions that increase prioritization of quality of life. Notably, activities that encourage distraction, respite, spontaneity, and the carrying on of memories and traditions may be critical for supporting the social, emotional, and mental health of people with progressive, potentially fatal illness.

## Supplementary Material

ocad234_Supplementary_Data

## Data Availability

The data gathered during this study is not publicly available and cannot be shared due to confidentiality, since participants are potentially identifiable from the information contained in the qualitative data. Furthermore, ethical restrictions from the consent process used with participants prevents data sharing and data requests. Any questions about this can be directed to the corresponding author.

## References

[1] World Health Organization. Chronic obstructive pulmonary disease (COPD). 2023. Accessed 16 August, 2023. http://www.who.int/mediacentre/factsheets/fs315/en/

[2] Benady S. The Human and Economic Burden of COPD: A Leading Cause of Hospital Admission in Canada. Canadian Thoracic Society; 2010.

[3] Public Health Agency of Canada. Life and Breath: Respiratory Disease in Canada (2007). Public Health Agency Canada; 2007.

[4] Global Initiative for Chronic Obstructive Lung Disease. Global strategy for prevention, diagnosis and management of COPD: 2023 report. 2023. Accessed October 15, 2023. https://goldcopd.org/wp-content/uploads/2023/03/GOLD-2023-ver-1.3-17Feb2023_WMV.pdf

[5] Gardiner C , Gott M, Payne S, et al Exploring the care needs of patients with advanced COPD: an overview of the literature. Respir Med. 2010;104(2):159-165. 19818590 10.1016/j.rmed.2009.09.007

[6] Molzahn A , Sheilds L, Antonio M, et al Ten minutes to midnight: a narrative inquiry of people living with dying with advanced COPD and their family members. Int J Qual Stud Health Well-Being. 2021;16(1):1893146. 10.1080/17482631.2021.1893146 PMC7946051 33683185

[7] Pavord ID , Jones PW, Burgel P-R, et al Exacerbations of COPD. COPD. 2016;11(Supl 1):21-30. 10.2147/COPD.S85978 PMC4764047 26937187

[8] Ekdahl A , Söderberg S, Rising‐Holmström M. Living with an ever‐present breathlessness: women’s experiences of living with chronic obstructive pulmonary disease stage III or IV. Scand J Caring Sci. 2022;36(4):1064-1073. 34008226 10.1111/scs.12998

[9] Barton C , Effing TW, Cafarella P. Social support and social networks in COPD: a scoping review. COPD J Chron Obstruct Pulmon Dis. 2015;12(6):690-702. 10.3109/15412555.2015.1008691 26263036

[10] Hill K , Geist R, Goldstein R, et al Anxiety and depression in end-stage COPD. Eur Respir J. 2008;31(3):667-677. 18310400 10.1183/09031936.00125707

[11] Gaffney AW , Himmelstein DU, Christiani DC, et al Socioeconomic inequality in respiratory health in the US From 1959 to 2018. JAMA Intern Med. 2021;181(7):968-976. 34047754 10.1001/jamainternmed.2021.2441 PMC8261605

[12] Mathioudakis AG , Ananth S, Vestbo J. Stigma: an unmet public health priority in COPD. Lancet Respir Med. 2021;9(9):955-956. 34197813 10.1016/S2213-2600(21)00316-7

[13] Breaden K , Collier A, Litster C, et al Stigma and the in (visible) perspectives and expectations of home oxygen therapy among people with chronic breathlessness syndrome: a qualitative study. Palliat Med. 2019;33(1):82-90. 30296930 10.1177/0269216318805621

[14] Woo S , Zhou W, Larson JL. Stigma experiences in people with chronic obstructive pulmonary disease: an integrative review. Int J Chron Obstruct Pulmon Dis. 2021;16(2021):1647-1659. 34113096 10.2147/COPD.S306874 PMC8187000

[15] Halding AG , Heggdal K, Wahl A. Experiences of self‐blame and stigmatisation for self‐infliction among individuals living with COPD. Scand Caring Sci. 2011;25(1):100-107. 10.1111/j.1471-6712.2010.00796.x 20534028

[16] Harrison SL , Robertson N, Apps L, et al “We are not worthy” — understanding why patients decline pulmonary rehabilitation following an acute exacerbation of COPD. Disabil Rehabil. 2015;37(9):750-756. 25009949 10.3109/09638288.2014.939770

[17] Johnson JL , Campbell AC, Bowers M, et al Understanding the social consequences of chronic obstructive pulmonary disease: the effects of stigma and gender. Proc Am Thoracic Soc. 2007;4(8):680-682. 10.1513/pats.200706-084SD 18073402

[18] Brighton LJ , Chilcot J, Maddocks M. Social dimensions of chronic respiratory disease: stigma, isolation, and loneliness. Curr Opin Support Palliat Care. 2022;16(4):195-202. 36102929 10.1097/SPC.0000000000000616

[19] Connolly M , Yohannes A. The impact of depression in older patients with chronic obstructive pulmonary disease and asthma. Maturitas. 2016;92:9-14. 27621232 10.1016/j.maturitas.2016.07.005

[20] Yohannes AM , Alexopoulos GS. Depression and anxiety in patients with COPD. Eur Respir Rev. 2014;23(133):345-349. 25176970 10.1183/09059180.00007813 PMC4523084

[21] Bourbeau J , Bhutani M, Hernandez P, et al CTS position statement: pharmacotherapy in patients with COPD—an update. Canadian J Resp Criti Care Sleep Med. 2017;1(4):222-241.

[22] Bourbeau J , Bhutani M, Hernandez P, et al Canadian Thoracic Society clinical practice guideline on pharmacotherapy in patients with COPD—2019 update of evidence. Can Respir J. 2019;3(4):210–232.

[23] Au DH , Feemster LC. Chronic obstructive pulmonary disease. Health Disparity Inequ. 2014;11(8):1250-1251. 10.1513/AnnalsATS.201408-406ED 25343193

[24] Gore JM , Brophy CJ, Greenstone MA. How well do we care for patients with end stage chronic obstructive pulmonary disease (COPD)? A comparison of palliative care and quality of life in COPD and lung cancer. Thorax. 2000;55(12):1000-1006. 11083884 10.1136/thorax.55.12.1000 PMC1745647

[25] Sadatsafavi M , editor. Burden of COPD in BC: where it stands, where it is heading, and how we can curb the curve. BC Ministry of Health—Research Rounds; February 28, 2017.

[26] Almagro P , Soriano JB. Underdiagnosis in COPD: a battle worth fighting. Lancet Respir Med. 2017;5(5):367-368. 28389226 10.1016/S2213-2600(17)30133-9

[27] Lamprecht B , Soriano JB, Studnicka M, et al Determinants of underdiagnosis of COPD in national and international surveys. Chest. 2015;148(4):971-985. 25950276 10.1378/chest.14-2535

[28] Martinez CH , Mannino DM, Jaimes FA, et al Undiagnosed obstructive lung disease in the United States. Associated factors and long-term mortality. Ann ATS. 2015;12(12):1788-1795. 10.1513/AnnalsATS.201506-388OC PMC4722830 26524488

[29] Evans J , Chen Y, Camp PG, et al Estimating the prevalence of COPD in Canada. Health Rep. 2014;25(3):3-11. 24648134

[30] Johnson KM , Khakban A, Bryan S, et al Healthcare system encounters before COPD diagnosis: a registry-based longitudinal cohort study. Thorax. 2020;75(2):108-115. 31704794 10.1136/thoraxjnl-2019-213554

[31] BCGuidelines.ca. Chronic obstructive pulmonary disease (COPD): diagnosis and management. 2020. Accessed October 14, 2023. https://www2.gov.bc.ca/assets/gov/health/practitioner-pro/bc-guidelines/copd_full_guideline.pdf

[32] Camp PG , Levy RD. A snapshot of chronic obstructive pulmonary disease in British Columbia and Canada. Br Columbia Med J. 2008;50(2):80. 10.1155/2008/120374 PMC2682163 19107241

[33] Gaffney AW , Hawks L, White AC, et al Health care disparities across the urban‐rural divide: a national study of individuals with COPD. J Rural Health. 2022;38(1):207-216. 33040358 10.1111/jrh.12525

[34] Lacasse Y , Cates CJ, McCarthy B, et al This Cochrane Review is closed: deciding what constitutes enough research and where next for pulmonary rehabilitation in COPD. Cochrane Database Syst Rev. 2015;(11):ED000107. 26593129 10.1002/14651858.ED000107 PMC10845864

[35] Rochester CL , Vogiatzis I, Holland AE, et al An official American Thoracic Society/European Respiratory Society policy statement: enhancing implementation, use, and delivery of pulmonary rehabilitation. Am J Respir Crit Care Med. 2015;192(11):1373-1386. 26623686 10.1164/rccm.201510-1966ST

[36] Brooks D , Sottana R, Bell B, et al Characterization of pulmonary rehabilitation programs in Canada in 2005. Can Respir J. 2007;14(2):87-92. 17372635 10.1155/2007/951498 PMC2676378

[37] Rawal H , Cornelison SD, Flynn SM, et al Will remotely based pulmonary rehabilitation water down its effectiveness? Life. 2021;11(11):1270. 34833145 10.3390/life11111270 PMC8625237

[38] Keating A , Lee A, Holland AE. What prevents people with chronic obstructive pulmonary disease from attending pulmonary rehabilitation? A systematic review. Chron Respir Dis. 2011;8(2):89-99. 21596892 10.1177/1479972310393756

[39] Hayton C , Clark A, Olive S, et al Barriers to pulmonary rehabilitation: characteristics that predict patient attendance and adherence. Respir Med. 2013;107(3):401-407. 23261311 10.1016/j.rmed.2012.11.016

[40] Burkow TM , Vognild LK, Johnsen E, et al Comprehensive pulmonary rehabilitation in home-based online groups: a mixed method pilot study in COPD. BMC Res Notes. 2015;8(1):1-11. 26651831 10.1186/s13104-015-1713-8 PMC4674913

[41] Siouta N , Heylen A, Aertgeerts B, et al Quality of life and quality of care in patients with advanced chronic heart failure (CHF) and advanced chronic obstructive pulmonary disease (COPD): implication for palliative care from a prospective observational study. Int J Hum Comput Interact. 2021;29(1):11-19.

[42] Thomashow BM , Mannino DM, Tal-Singer R, et al A rapidly changing understanding of COPD: world COPD Day from the COPD Foundation. Am J Physiol Lung Cell Mol Physiol. 2021;321(5):L983-L987. 34612086 10.1152/ajplung.00400.2021

[43] Klasnja P , Pratt W. Healthcare in the pocket: mapping the space of mobile-phone health interventions. J Biomed Inform. 2012;45(1):184-198. 21925288 10.1016/j.jbi.2011.08.017 PMC3272165

[44] Demiris G. Consumer health informatics: past, present, and future of a rapidly evolving domain. Yearb Med Inform. 2016;25(S 01):S42-S47. 10.15265/IYS-2016-s005 PMC5171509 27199196

[45] American Medical Informatics Association. Consumer health informatics. 2023. Accessed August 15, 2023. https://amia.org/about-amia/why-informatics/informatics-research-and-practice

[46] Faiola A , Holden RJ. Consumer health informatics: empowering healthy-living-seekers through mHealth. Prog Cardiovasc Dis. 2017;59(5):479-486. 28038910 10.1016/j.pcad.2016.12.006

[47] Gibbons MC , Wilson RF, Samal L, et al Consumer health informatics: results of a systematic evidence review and evidence based recommendations. Behav Med Pract Policy Res. 2011;1(1):72-82. 10.1007/s13142-011-0016-4 PMC3717687 24073033

[48] Lai AM , Hsueh P-Y, Choi YK, et al Present and future trends in consumer health informatics and patient-generated health data. Yearb Med Inform. 2017;26(01):152-159. 29063559 10.15265/IY-2017-016 PMC6239232

[49] Lewis D , Chang BL, Friedman CP. Consumer Health Informatics. Consumer Health Informatics: Informing Consumers and Improving Health Care. Springer; 2005:1-7.

[50] Nguyen HQ , Donesky D, Reinke LF, et al Internet-based dyspnea self-management support for patients with chronic obstructive pulmonary disease. J Pain Symptom Manage. 2013;46(1):43-55. 23073395 10.1016/j.jpainsymman.2012.06.015 PMC3548968

[51] McCabe C , McCann M, Brady AM. Computer and mobile technology interventions for self‐management in chronic obstructive pulmonary disease. Cochrane Database Syst Rev. 2017;5(5):CD011425. 28535331 10.1002/14651858.CD011425.pub2 PMC6481891

[52] Talboom-Kamp E , Verdijk N, Kasteleyn M, et al The effect of integration of self-management web platforms on health status in chronic obstructive pulmonary disease management in primary care (e-Vita stud): interrupted time series design. J Med Internet Res. 2017;19(8):e291. 28814380 10.2196/jmir.8262 PMC5577456

[53] Watson A , Wilkinson TM. Digital healthcare in COPD management: a narrative review on the advantages, pitfalls, and need for further research. Ther Adv Respir Dis. 2022;16:175346662210754. 17534666221075493. 10.1177/17534666221075493 PMC8894614 35234090

[54] Bosnic-Anticevich S , Bakerly ND, Chrystyn H, et al Advancing digital solutions to overcome longstanding barriers in asthma and COPD management. Patient Prefer Adherence. 2023;17(2023):259-272. 36741814 10.2147/PPA.S385857 PMC9891071

[55] Cravo A , Attar D, Freeman D, et al The importance of self-management in the context of personalized care in COPD. Int J Chron Obstruct Pulmon Dis. 2022;17(2022):231-243. 35095272 10.2147/COPD.S343108 PMC8791295

[56] Smalley KR , Aufegger L, Flott K, et al Can self-management programmes change healthcare utilisation in COPD?: a systematic review and framework analysis. Patient Educ Couns. 2021;104(1):50-63. 32912809 10.1016/j.pec.2020.08.015 PMC7762718

[57] Quach S , Benoit A, Oliveira A, et al Features and characteristics of publicly available mHealth apps for self-management in chronic obstructive pulmonary disease. Digital Health. 2023;9:205520762311670. 10.1177/20552076231167007 PMC10102951 37065541

[58] Sobnath DD , Philip N, Kayyali R, et al Features of a mobile support app for patients With chronic obstructive pulmonary disease: literature review and current applications. JMIR Mhealth Uhealth. 2017;5(2):e17. 28219878 10.2196/mhealth.4951 PMC5339437

[59] Ding H , Fatehi F, Maiorana A, et al Digital health for COPD care: the current state of play. J Thorac Dis. 2019;11(S17):S2210-S2220. 31737348 10.21037/jtd.2019.10.17 PMC6831928

[60] Silva J , Hipólito N, Machado P, et al Technological features of smartphone apps for physical activity promotion in patients with COPD: a systematic review. Pulmonology. 2023. 10.1016/j.pulmoe.2023.06.005 37394341

[61] Kooij L , Vos PJ, Dijkstra A, et al Effectiveness of a mobile health and self-management app for high-risk patients with chronic obstructive pulmonary disease in daily clinical practice: mixed methods evaluation study. JMIR Mhealth Uhealth. 2021;9(2):e21977. 33538699 10.2196/21977 PMC7892284

[62] Greenhalgh T. Patient and public involvement chronic illness: beyond the expert patient. BMJ. 2009;338(1):b49. 19223339 10.1136/bmj.b49

[63] Bos-Touwen I , Schuurmans M, Monninkhof EM, et al Patient and disease characteristics associated with activation for self-management in patients with diabetes, chronic obstructive pulmonary disease, chronic heart failure and chronic renal disease: a cross-sectional survey study. PLoS One. 2015;10(5):e0126400. 25950517 10.1371/journal.pone.0126400 PMC4423990

[64] Jonsdottir H. Self‐management programmes for people living with chronic obstructive pulmonary disease: a call for a reconceptualisation. J Clin Nurs. 2013;22(5-6):621-637. 23398312 10.1111/jocn.12100

[65] Shaw G , Whelan M, Armitage L, et al Are COPD self-management mobile applications effective? A systematic review and meta-analysis. NPJ Prim Care Respir Med. 2020;30(1):11. 32238810 10.1038/s41533-020-0167-1 PMC7113264

[66] Sunjaya AP , Sengupta A, Martin A, et al Efficacy of self-management mobile applications for patients with breathlessness: systematic review and quality assessment of publicly available applications. Respir Med. 2022;201:106947. 36037561 10.1016/j.rmed.2022.106947

[67] Scott IA , Scuffham P, Gupta D, et al Going digital: a narrative overview of the effects, quality and utility of mobile apps in chronic disease self-management. Aust Health Review. 2020;44(1):62-82. 10.1071/AH18064 30419185

[68] Cucciniello M , Petracca F, Ciani O, et al Development features and study characteristics of mobile health apps in the management of chronic conditions: a systematic review of randomised trials. NPJ Digit Med. 2021;4(1):144. 34611287 10.1038/s41746-021-00517-1 PMC8492762

[69] Wagner EH , Austin BT, Davis C, et al Improving chronic illness care: translating evidence into action. Health Aff. 2001;20(6):64-78. 10.1377/hlthaff.20.6.64 11816692

[70] Nunes F , Verdezoto N, Fitzpatrick G, et al Self-care technologies in HCI: trends, tensions, and opportunities. ACM Trans Comput Hum Interact. 2015;22(6):1-45.

[71] Antonio MG , Petrovskaya O, Lau F. The state of evidence in patient portals: umbrella review. J Med Internet Res. 2020;22(11):e23851. 33174851 10.2196/23851 PMC7688386

[72] Adhikary D , Barman S, Ranjan R. Internet-based cognitive behavioural therapy for individuals with depression and chronic health conditions: a systematic review. Cureus. 2023;15(4):e37822. 10.7759/cureus.37822 PMC10197913 37213982

[73] Merolli M , Gray K, Martin-Sanchez F. Health outcomes and related effects of using social media in chronic disease management: a literature review and analysis of affordances. J Biomed Inform. 2013;46(6):957-969. 23702104 10.1016/j.jbi.2013.04.010

[74] Moorhead SA , Hazlett DE, Harrison L, et al A new dimension of health care: systematic review of the uses, benefits, and limitations of social media for health communication. J Med Internet Res. 2013;15(4):e85. 23615206 10.2196/jmir.1933 PMC3636326

[75] Veinot TC. “We have a lot of information to share with each other”: understanding the value of peer-based health information exchange. Inform Res. 2010;15(4):15-14.

[76] Veinot TC , Ancker JS, Cole-Lewis H, et al Leveling up: on the potential of upstream health informatics interventions to enhance health equity. Med Care. 2019;57(Suppl 2):S108-S114. 31095048 10.1097/MLR.0000000000001032

[77] Solar O , Irwin A. A Conceptual Framework for Action on the Social Determinants of Health: Social Determinants of Health Discussion Paper 2 (Policy and Practice). World Health Organization; 2010.

[78] Parker A , Kantroo V, Lee HR, et al, eds. Health promotion as activism: building community capacity to effect social change. In: *Proceedings of the SIGCHI Conference on Human Factors in Computing Systems*, Austin, TX. 2012.

[79] Mertens D. Transformative paradigm: mixed methods and social justice. J Mix Methods Res. 2007;1(3):212-225.

[80] Mertens D. Mixed methods and the politics of human research: the transformative-emancipatory perspective. In: Tashakkori ATC, ed. Handbook of Mixed Methods in Social and Behavioral Research. Thousand Oaks. SAGE Publications; 2003:135-164.

[81] Mertens D. What does a transformative lens bring to credible evidence in mixed methods evaluations? New Direct Eval. 2013;2013(138):27-35.

[82] Mertens D. Transformative Research and Evaluation. Guilford Press; 2008.

[83] Mertens D , Bledsoe K, Sullivan M, et alUtilization of mixed methods for transformative purposes. In: Tashakkori A, Teddlie C, eds. SAGE Handbook of Mixed Methods in Social & Behavioral Research. 2nd ed. SAGE Publications; 2010:193-214.

[84] Mertens D , Hesse‐Biber S. Mixed methods and credibility of evidence in evaluation. New Direc Eval. 2013;2013(138):5-13.

[85] Mertens DM. Transformative mixed methods: addressing inequities. Am Behav Sc. 2012;56(6):802-813.

[86] Mertens DM. Divergence and Mixed Methods. Sage Publications; 2010:3-5.

[87] Mertens DM , Hesse-Biber S. Triangulation and Mixed Methods Research: Provocative Positions. Sage Publications; 2012;6(2):75-79.

[88] Denzin NK. Triangulation 2.0. J Mix Methods Res. 2012;6(2):80-88.

[89] Veinot TC , Clarke PJ, Romero DM, et al Equitable research PRAXIS: a framework for health informatics methods. Yearb Med Inform. 2022;31(01):307-316. 36463889 10.1055/s-0042-1742542 PMC9719773

[90] Mertens DM. Transformative research methods to increase social impact for vulnerable groups and cultural minorities. Int J Qual Methods. 2021;20.

[91] Unertl KM , Schaefbauer CL, Campbell TR, et al Integrating community-based participatory research and informatics approaches to improve the engagement and health of underserved populations. J Am Med Inform Assoc. 2016;23(1):60-73. 26228766 10.1093/jamia/ocv094 PMC4713901

[92] Van Bel DT , Smolders K, IJsselsteijn WA, et al Social connectedness: concept and measurement. Intell Environ. 2009;2:67-74.

[93] Estoque RC , Togawa T, Ooba M, et al A review of quality of life (QOL) assessments and indicators: towards a "QOL-Climate" assessment framework. Ambio. 2019;48(6):619-638. 30206898 10.1007/s13280-018-1090-3 PMC6486941

[94] Farmer P. Pathologies of Power: Health, Human Rights, and the New War on the Poor. University of California Press; 2004.

[95] Fiske A , Galasso I, Eichinger J, et al The second pandemic: examining structural inequality through reverberations of COVID-19 in Europe. Soc Sci Med. 2022;292:114634. 34883310 10.1016/j.socscimed.2021.114634 PMC8648175

[96] Centers for Disease Control and Prevention. The COPD national action plan. 2021.

[97] Suurmond J , Lanting K, de Voogd X, et al Twelve tips to teach culturally sensitive palliative care. Med Teach. 2021;43(7):845-850. 33070696 10.1080/0142159X.2020.1832650

[98] Alwashmi MF , Fitzpatrick B, Davis E, et al Perceptions of health care providers regarding a mobile health intervention to manage chronic obstructive pulmonary disease: qualitative study. JMIR Mhealth Uhealth. 2019;7(6):e13950. 31199330 10.2196/13950 PMC6592475

[99] Alwashmi MF , Fitzpatrick B, Farrell J, et al Perceptions of patients regarding mobile health interventions for the management of chronic obstructive pulmonary disease: mixed methods study. JMIR Mhealth Uhealth. 2020;8(7):e17409. 32706697 10.2196/17409 PMC7413289

[100] Antonio MG , Petrovskaya O, Lau F. Is research on patient portals attuned to health equity? A scoping review. J Am Med Inform Assoc. 2019;26(8-9):871-883. 31066893 10.1093/jamia/ocz054 PMC7647227

[101] Hodson M , Roberts CM, Andrew S, et al Development and first validation of a patient-reported experience measure in chronic obstructive pulmonary disease (PREM-C9). Thorax. 2019;74(6):600-603. 31028236 10.1136/thoraxjnl-2018-211732

[102] GlaxoSmithKline. COPD assessment test: healthcare professional user guide. 2018. Accessed 10 October, 2023. https://www.catestonline.org/

[103] Hahn EA , Beaumont JL, Pilkonis PA, et al The PROMIS satisfaction with social participation measures demonstrated responsiveness in diverse clinical populations. J Clin Epidemiol. 2016;73:135-141. 26931288 10.1016/j.jclinepi.2015.08.034 PMC4902758

[104] Hodson M. Development of a Patient Reported Experience Measure in Chronic Obstructive Pulmonary Disease (COPD). University of Portsmouth; 2014.

[105] Deshpande PR , Rajan S, Sudeepthi BL, et al Patient-reported outcomes: a new era in clinical research. Perspect Clin Res. 2011;2(4):137. 22145124 10.4103/2229-3485.86879 PMC3227331

[106] U.S. Department of Health and Human Services, Food and Drug Administration. Guidance for industry patient-reported outcome measures: use in medical product development to support labeling claims USA. 2006. Accessed 10 October, 2023. https://link.springer.com/article/10.1186/1477-7525-4-79 10.1186/1477-7525-4-79 PMC1629006 17034633

[107] National Institutes of Health. Patient-reported outcomes measurement information system (PROMIS). 2023. Accessed 18 October, 2023. https://commonfund.nih.gov/promis/index

[108] U.S. Department of Health and Human Services, Food and Drug Administration, Center for Drug Evaluation and Research (CDER), et al *Guidance for Industry Patient-Reported Outcome Measures: Use in Medical Product Development to Support Labeling Claims*. Food and Drug Administration, Center for Drug Evaluation and Research (CDER), Center for Biologics Evaluation and Research (CBER), Center for Devices and Radiological Health (CDRH); 2009.

[109] Afroz N , Gutzwiller FS, Mackay AJ, et al Patient-reported outcomes (PROs) in COPD clinical trials: trends and gaps. Int J Chron Obstruct Pulmon Dis. 2020;15:1789-1800. 32801678 10.2147/COPD.S235845 PMC7398869

[110] Collins K. Advanced sampling designs in mixed research: current practices and emerging trends in the social and behavioral sciences. In: Tashakkori A, Teddlie C, eds. SAGE Handbook of Mixed Methods in Social & Behavioral Research. SAGE Publications; 2010:353-378.

[111] Onwuegbuzie AJ , Collins KM. A typology of mixed methods sampling designs in social science research. Qual Rep. 2007;12(2):281-316.

[112] Atlasti.com. ATLAS.ti. 2023. Accessed 14 August, 2023. https://atlasti.com/

[113] Saldaña J. The Coding Manual for Qualitative Researchers. 3rd ed. SAGE Publications; 2016.

[114] Bazeley P. Integrating Analyses into Mixed Methods Research. SAGE Publishications; 2018.

[115] Guetterman TC , Fetters MD, Creswell JW. Integrating quantitative and qualitative results in health science mixed methods research through joint displays. Ann Fam Med. 2015;13(6):554-561. 26553895 10.1370/afm.1865 PMC4639381

[116] Vehling S , Kissane DW. Existential distress in cancer: alleviating suffering from fundamental loss and change. Psychooncology. 2018;27(11):2525-2530. 30307088 10.1002/pon.4872

[117] Lupton D. Health promotion in the digital era: a critical commentary. Health Promot Int. 2014;30(1):174-183. 25320120 10.1093/heapro/dau091

[118] Claisse C , Kasadha B, Stumpf S, et al, eds. Investigating daily practices of self-care to inform the design of supportive health technologies for living and ageing well with HIV. In: *Proceedings of the 2022 CHI Conference on Human Factors in Computing Systems*, New Orleans, LA. 2022.

[119] Rao D , Elshafei A, Nguyen M, et al A systematic review of multi-level stigma interventions: state of the science and future directions. BMC Med. 2019;17(1):1-11. 30770756 10.1186/s12916-018-1244-y PMC6377735

[120] Iyer AS , Sullivan DR, Lindell KO, et al The role of palliative care in COPD. Chest. 2022;161(5):1250-1262. 34740592 10.1016/j.chest.2021.10.032 PMC9131048

[121] Guerrero Z , Iruretagoyena B, Parry S, et al Anti-stigma advocacy for health professionals: a systematic review. J Ment. 2023:1-21. 10.1080/09638237.2023.2182421 PMC10173949 36919957

[122] Fang ML , Sixsmith J, Sinclair S, et al A knowledge synthesis of culturally- and spiritually-sensitive end-of-life care: findings from a scoping review. BMC Geriatr. 2016;16(1):107. 27193395 10.1186/s12877-016-0282-6 PMC4872365

[123] Ingle MP , Valdovinos C, Ford KL, et al Patient portals to support palliative and end-of-life care: scoping review. J Med Internet Res. 2021;23(9):e28797. 34528888 10.2196/28797 PMC8485198

[124] Uhler LM , Figueroa REP, Dickson M, et al Informed together: usability evaluation of a web-based decision aid to facilitate shared advance care planning for severe chronic obstructive pulmonary disease. JMIR Human Factors. 2015;2(1):e3842. 10.2196/humanfactors.3842 PMC4797670 27025896

[125] Nunes F. From medicalized to mundane self-care technologies. Interactions. 2019;26(3):67-69.

[126] Wolf CT , Veinot TC. Struggling for space and finding my place: an interactionist perspective on everyday use of biomedical information. J Assoc Inform Sci Technol. 2015;66(2):282-296.

[127] Meehan E , Foley T, Kelly C, et al Advance care planning for individuals with chronic obstructive pulmonary disease: a scoping review of the literature. J Pain Symptom Manage. 2020;59(6):1344-1361. 31837455 10.1016/j.jpainsymman.2019.12.010

[128] World Health Organization. Policy Brief on Integrating Rehabilitation into Palliative Care Services. World Health Organization, Regional Office for Europe; 2023.

[129] Chang H , Do Y, Ahn J. Digital storytelling as an intervention for older adults: a scoping review. Int J Environ Res Public Health. 2023;20(2):1344. 36674100 10.3390/ijerph20021344 PMC9859096

[130] Hausknecht S , Vanchu-Orosco M, Kaufman D. Digitising the wisdom of our elders: connectedness through digital storytelling. Ageing Soc. 2019;39(12):2714-2734.

[131] Rios Rincon AM , Miguel Cruz A, Daum C, et al Digital storytelling in older adults with typical aging, and with mild cognitive impairment or dementia: a systematic literature review. J Appl Gerontol. 2022;41(3):867-880. 34009053 10.1177/07334648211015456 PMC8848055

[132] de la Hera Conde-Pumpido T , Sanz CS. The role of spontaneous digital play during young patients’ cancer treatment. Media Commun. 2021;9(1):39-48.

[133] Ma R-C , Yin Y-Y, Wang Y-Q, et al Effectiveness of cognitive behavioural therapy for chronic obstructive pulmonary disease patients: a systematic review and meta‐analysis. Complement Ther Clin Pract. 2020;38:101071. 31743870 10.1016/j.ctcp.2019.101071

[134] Thach KS , Lederman R, Waycott J, eds. How older adults respond to the use of virtual reality for enrichment: a systematic review. In: *Proceedings of the 32nd Australian Conference on Human-Computer Interaction*, Sydney, Australia. 2020.

[135] Baker S , Kelly RM, Waycott J, et al School’s back: scaffolding reminiscence in social virtual reality with older adults. Proc ACM Hum Comput Interact. 2021;4(CSCW3):1-25.

[136] Padamsee TJ. The politics of prevention: lessons from the neglected history of US HIV/AIDS policy. J Health Polit Policy Law. 2017;42(1):73-122. 27729443 10.1215/03616878-3702782

[137] Braun S. The history of breast cancer advocacy. Breast J. 2003;9(s2):S101-S103. 12713506 10.1046/j.1524-4741.9.s2.13.x

[138] Hoffman BS , Ellen, Stovall E. Survivorship perspectives and advocacy. J Clin Oncol. 2006;24(32):5154-5159. 17093279 10.1200/JCO.2006.06.5300

[139] Andalibi N , Forte A, eds. Announcing pregnancy loss on Facebook: a decision-making framework for stigmatized disclosures on identified social network sites. In: *Proceedings of the 2018 CHI Conference on Human Factors in Computing Systems*, Montréal, Canada. 2018.

[140] Betton V , Borschmann R, Docherty M, et al The role of social media in reducing stigma and discrimination. Br J Psychiatry. 2015;206(6):443-444. 26034176 10.1192/bjp.bp.114.152835

[141] BC Lung Foundation. COPD support group. 2023. Accessed 6 August, 2023. www.bclungfoundation.ca

[142] Kaptein AA , Fischer MJ, Scharloo M. Self-management in patients with COPD: theoretical context, content, outcomes, and integration into clinical care. Int J Chron Obstruct Pulmon Dis. 2014;9(2014):907-917. 25214777 10.2147/COPD.S49622 PMC4159069

[143] Glasgow RE , Orleans CT, Wagner EH. Does the chronic care model serve also as a template for improving prevention? Milbank Q. 2001;79(4):579-612. 11789118 10.1111/1468-0009.00222 PMC2751207

[144] Coleman K , Austin BT, Brach C, et al Evidence on the chronic care model in the new millennium. Health Aff. 2009;28(1):75-85. 10.1377/hlthaff.28.1.75 PMC5091929 19124857

[145] Moullec G , Ninot G. An integrated programme after pulmonary rehabilitation in patients with chronic obstructive pulmonary disease: effect on emotional and functional dimensions of quality of life. Clin Rehabil. 2010;24(2):122-136. 20026578 10.1177/0269215509346088

[146] Cox NS , Khor YH. Telerehabilitation in pulmonary diseases. Curr Opin Pulm Med. 2023;29(4):313-321. 37132293 10.1097/MCP.0000000000000962

[147] Taylor A , Godden D, Aitken A, et al, eds. Delivering group-based services to the home via the Internet: maximising clinical and social benefits. In: *2011 5th International Conference on Pervasive Computing Technologies for Healthcare (PervasiveHealth) and Workshops*, Dublin, Ireland. IEEE; 2011.

[148] Tendedez H , Ferrario M-A, McNaney R, et al, eds. Respiratory self-care: identifying current challenges and future potentials for digital technology to support people with chronic respiratory conditions. In: *Proceedings of the 13th EAI International Conference on Pervasive Computing Technologies for Healthcare*, Trento, Italy. 2019.

[149] Pols J. Knowing patients: turning patient knowledge into science. Sci Technol Human Values. 2014;39(1):73-97.

[150] May CR , Cummings A, Myall M, et al Experiences of long-term life-limiting conditions among patients and carers: what can we learn from a meta-review of systematic reviews of qualitative studies of chronic heart failure, chronic obstructive pulmonary disease and chronic kidney disease? BMJ Open. 2016;6(10):e011694. 10.1136/bmjopen-2016-011694 PMC5073552 27707824

[151] Huffziger S , Kuehner C. Rumination, distraction, and mindful self-focus in depressed patients. Behav Res Ther. 2009;47(3):224-230. 19166993 10.1016/j.brat.2008.12.005

[152] Fulford H , McSwiggan L, Kroll T, et al Exploring the use of mobile information and communication technologies by people with mood disorders. Int J Mental Health Nurs. 2019;28(6):1268-1277. 10.1111/inm.12632 31325245

[153] Nurain N , Chung C-F, eds. “I left my legacy, told my story”: understanding older adults’ tracking practices to promote active aging. In: *Proceedings of the 2023 ACM Designing Interactive Systems Conference*, 2023.

[154] Krzeczkowska A , Spalding DM, McGeown WJ, et al A systematic review of the impacts of intergenerational engagement on older adults’ cognitive, social, and health outcomes. Ageing Res Rev. 2021;71:101400. 34237435 10.1016/j.arr.2021.101400

[155] Molzahn AE , Sheilds L, Bruce A, et al Life and priorities before death: a narrative inquiry of uncertainty and end of life in people with heart failure and their family members. Eur J Cardiovasc Nurs. 2020;19(7):629-637. 32340476 10.1177/1474515120918355

[156] Alexandrakis D , Chorianopoulos K, Tselios N. Older adults and web 2.0 storytelling technologies: disability and rehabilitation probing the technology acceptance model through an age-related perspective. Int J Hum Comput Interact. 2020;36(17):1623-1635.

[157] Stanley S , Higginbotham K, Finucane A, et al A grounded theory study exploring palliative care healthcare professionals’ experiences of managing digital legacy as part of advance care planning for people receiving palliative care. Palliat Med. 2023;37(9):1424-1433. 37609897 10.1177/02692163231194198 PMC10566216

[158] Doyle DT , Brubaker JR. Digital legacy: a systematic literature review. Proc ACM Hum Comput Interact. 2023;7(CSCW2):1-26.

[159] Sala G , Jopp D, Gobet F, et al The impact of leisure activities on older adults’ cognitive function, physical function, and mental health. PLoS One. 2019;14(11):e0225006. 31703115 10.1371/journal.pone.0225006 PMC6839878

[160] Ryu J , Heo J. Relationships between leisure activity types and well-being in older adults. Leis Stud. 2018;37(3):331-342.

[161] Paterson BL. The shifting perspectives model of chronic illness. J Nursing Scholarship. 2001;33(1):21-26. 10.1111/j.1547-5069.2001.00021.x 11253576

[162] Maietta JT. Integrating illness management into identity verification processes. Qual Health Res. 2021;31(2):254-270. 33135569 10.1177/1049732320966582

[163] Ahlström G. Experiences of loss and chronic sorrow in persons with severe chronic illness. J Clin Nurs. 2007;16(3a):76-83. 17518872 10.1111/j.1365-2702.2006.01580.x

[164] Hänninen R , Korpela V, Pajula L. The paradoxes and pragmatics of digital leisure in later life. Leis Stud. 2023;1-14.

[165] Hewes J. Seeking balance in motion: the role of spontaneous free play in promoting social and emotional health in early childhood care and education. Children. 2014;1(3):280-301. 27417480 10.3390/children1030280 PMC4928743

[166] Toepoel V. Ageing, leisure, and social connectedness: how could leisure help reduce social isolation of older people? Soc Indic Res. 2013;113(1):355-372. 23874058 10.1007/s11205-012-0097-6 PMC3696179

[167] Rivera-Torres S , Mpofu E, Jean Keller M, et al Older adults’ mental health through leisure activities during COVID-19: a scoping review. Gerontol Geriatr Med. 2021;7:233372142110367. 23337214211036776. 10.1177/23337214211036776 PMC8361539 34395816

