# Correction to: From illness management to quality of life: rethinking consumer health informatics opportunities for progressive, potentially fatal illnesses

**DOI:** 10.1093/jamia/ocae048

**Published:** 2024-03-28

This is a correction to: Marcy G Antonio, Tiffany C Veinot, From illness management to quality of life: rethinking consumer health informatics opportunities for progressive, potentially fatal illnesses, Journal of the American Medical Informatics Association, Volume 31, Issue 3, March 2024, Pages 674–691, https://doi.org/10.1093/jamia/ocad234

Upon original publication, the supplementary data files accompanying this article were incorrectly labelled in the Supplementary data section.

This has been corrected.

In addition, in the title of Multimedia Appendix SB, "All Participants" has been changed to read "Adopters".

Finally, the supplementary data file entitled “Multimedia Appendix SC_ Qualitative Data from Participants Living with a Progressive, Potentially Fatal Lung Condition.pdf” was inadvertently omitted and has now been added to the Supplementary data section.

