# Supplementary material for: From illness management to quality of life: rethinking consumer health informatics opportunities for progressive, potentially fatal illnesses
Source: J Am Med Inform Assoc. 2023 Dec 22;31(3):674–91. doi: 10.1093/jamia/ocad234 (PMC10873853; doi:10.1093/jamia/ocad234)
Supplement: ocad234_Supplementary_Data [file ocad234_supplementary_data.zip › ocad234_Supplementary_Data/Multimedia Appendix SA_Participant Characteristics (15).pdf]

Multimedia Appendix SA: Characteristics of Participants

Table 1: Participant Demographics

	**All Stages COPD participants (n=77)**	**Interviews COPD participants (n=15)**	**All Stages Non-COPD participants (n=6)**
**Age**			
Under 59	3 (3.9%)	0	1 (16.7%)
60-69	16 (20.8%)	5 (33.3%)	2 (33.3%)
70-79	39 (50.6%)	7 (46.7%)	2 (33.3%)
80-89	18 (23.4%)	3 (20.0%)	1 (16.7%)
Over 90	1 (1.3%)	0	0
**Gender**			
Man	29 (37.7%)	6 (40.0%)	2 (33.3%)
Woman	47 (61.0 %)	9 (60.0%)	4 (66.7%)
Cisgender	76 (100%)	15 (100%)	6 (100%)
Not provided	1	0	0
**Cultural background** (can choose more than one)			
White or European descent	64 (81.9%)	12 (80.0%)	3 (50%)
Canadian	11 (14.5%)	3 (20.0%)	2 (33.3%)
Indigenous	2 (2.4%)	2 (13.3%)	0
Preferred not to answer	8 (10.8%)	0	0
Not provided	0	0	1 (16.7%)
**Employment status**			
Retired	62 (80.5%)	11 (73.3%)	5 (83.3%)
Long-term disability or employment insurance	9 (11.7%)	3 (20.0%)	0
Home-maker	1 (1.2%)	0	0
Employed or self-employed	5 (5.2%)	1 (6.7%)	1(16.7%)
**Difficulties making ends meet**			
Never	25 (32.5%)	9 (60.0%)	2 (33.3%)
Rarely or sometimes	36 (46.8%)	5 (33.4%)	2 (33.3%)
Very often or always	9 (11.7%)	0	
Prefer not to answer	5 (6.5%)	1 (6.7%)	
Not provided	2 (2.6%)	1	2 (33.3%)
**Education**			
Some elementary or high school	12 (15.6%)	1 (6.7%)	0
High school graduate	16 (20.8%)	2 (13.3%)	0
Some college or trade school	13 (16.9%)	5 (33.3%)	1 (16.7%)
College or trade graduate	18 (23.4%)	2 (13.3%)	1 (16.7%)
Bachelor degree	6 (7.8%)	0 (0%)	3 (50.05)
Graduate or professional degree	9 (11.7%)	4 (26.6%)	1 (16.7%)
Prefer not to answer	1 (1.2%)	1 (6.7%)	0
Another response	1	0	0

**Geographic Location**			
Metropolitan (over 1 million)	9 (10.8%)	2 (13.3%)	0
Large urban population (over 100,000)	31 (38.6%)	7 (46.7%)	5 (83.3%)
Medium population centres (between 30,000 and 99,999)	27 (37.3%)	5 (33.3%)	1 (16.7%)
Small population centre (between 1,000 and 29,999)	5 (7.2%)	1 (6.7%)	0
Not provided	5	0	0
**Living situation**			
Alone	32 (41.6%)	7 (46.7%)	2 (33.3%)
Spouse or partner	40 (51.9%)	6 (40.0%)	2 (33.3%)
Parent, child or sibling	5 (6.5%)	0	2 (33.3%)
Grandchild(ren)	2 (2.6%)	2 (13.3%)	
Another option not listed (care facility, dog)	4 (5.2%)	0	0
**Marital status**			
Single	8 (10.4%)	2 (13.3%)	1 (16.7%)
Married or common-law	40 (51.9%)	7 (46.7%)	3 (50.0%)
Separated, divorced	10 (13.0%)	3 (20.0%)	1 (16.7%)
Widowed	16 (19.5%)	3 (20.0%)	0
Not provided	1	0	1 (16.7%)

Table 2: Results from Measures on Patient-Reported Outcomes (PROs)

**All survey participants** (n=80)	**All COPD survey participants** (n=68-72)	**Interviews COPD participants** (n=13)	**All Stages Non-COPD participants** (n=3-4)
**COPD-Assessment Test (CAT)** [1]			
M=19.67; SD=6.49	M=19.38; SD=6.41	M=17.69; SD=5.41	M=24.50; SD=6.76
**PROMIS Satisfaction in Social Roles and Activities** [2]			
M=7.66; SD=4.52	M=7.78; SD=4.45	M=7.62; SD=4.59	M=5.50; SD=6.03
**Patient-Reported Experience Measure – COPD (PREM-C9)** [3]			
M=16.08; SD=7.90	M=16.24; SD=7.85	M=16.31; SD=8.64	M=15.67; SD=10.1

[1] A high severity score (>20) on the COPD Assessment Test (CAT)[(99)] is interpreted as COPD is impacting the lives of people to the point where the symptoms prevent people from doing most their activities and having a restful sleep, and people may have fear and anxiety in not feeling in control of their illness.
[2] Unlike the other patient-reported measure, PROMIS Satisfaction with Social Roles and Activities,[(100)] has positive wording such that the overall score is in opposite direction to the PREM-C9 and CAT (i.e., a higher score indicates a stronger overall satisfaction with social roles and activities).
[3] There currently is not a validated guide for how to interpret results for PREM-C9.[(98, 101)] '0' on a question indicates a good experience and '5' indicates a bad experience. A score of 45 indicates highest overall score, and poorest experience of living with COPD.
