# Supplementary material for: From illness management to quality of life: rethinking consumer health informatics opportunities for progressive, potentially fatal illnesses
Source: J Am Med Inform Assoc. 2023 Dec 22;31(3):674–91. doi: 10.1093/jamia/ocad234 (PMC10873853; doi:10.1093/jamia/ocad234)
Supplement: ocad234_Supplementary_Data [file ocad234_supplementary_data.zip › ocad234_Supplementary_Data/Multimedia Appendix SB_Reporting of Use of Digital Technologies Amongst ....pdf]

Multimedia Appendix SB: Reporting of Use of Digital Technologies Amongst Adopters

Table 1: Frequency of Using Digital Technologies for Social Connections

**Type of Connection**	**Frequency Reported**			
	**Not at all**	**A little bit/Somewhat**	**Quite a bit/ Very much**	**Did not answer**
Other people living with COPD	53.6 % (37/69)	33.3 % (23/69)	13.0 % (9/69)	2
My family	10.6% (7/66)	30.3% (20/66)	59.0% (39/66)	5
My face-to-face friends	17.6% (12/68)	42.6% (29/68)	39.7% (27/68)	2
My online friends	30.4% (21/69)	34.8% (24/69)	34.8% (24/69)	2
My local community	34.3% (23/67)	50.7% (34/67)	14.9% (10/67)	4
My religious/spiritual association	71.2% (47/66)	18.2% (12/66)	10.6% (7/66)	5
People who I have lost contact with	45.5% (30/66)	51.5% (34/66)	3.0% (2/66)	5
My health professionals (e.g., doctor, nurse, respiratory therapist)	47.1% (32/68)	48.5% (33/68)	4.4% (3/68)	3
Other people who support my care	53.1% (34/64)	34.3% (22/64)	12.5% (8/64)	7
Emergency services	71.2% (42/59)	22.0% (13/59)	6.8% (4/59)	12

Table 2: Frequency of Using Digital Technologies for Illness-Related Activities

Activity	Frequency Reported			
	Not at all	A little bit/Somewhat	Quite a bit/ Very much	Did not answer
Find out statistics	37.1% (26/70)	47.1% (33/70)	15.7% (11/70)	1
Learn about symptoms	15.9% (11/69)	50.8% (35/69)	33.3% (23/69)	2
Learn about medications and treatments	15.9% (11/69)	59.4% (41/69)	24.6% (17/69)	2
Breathing exercises	42.0% (29/69)	40.6 % (28/69)	17.4 % (12/69)	2
Search for programs and services	27.9% (19/68)	50% (34/68)	22.1% (15/68)	3
Participate in programs to manage my illness	43.5% (30/69)	30.4% (21/69)	26% (18/69)	2
Participate in support groups	41.2% (28/68)	32.3% (22/68)	26.5% (18/68)	3
Discuss my illness experiences with others	44.8% (30/67)	43.3 % (29/67)	11.9 % (8/67)	4
Record information discussed with my health professional (e.g., doctor, nurse, respiratory therapist)	52.2% (35/67)	37.3% (25/67)	10.4% (7/67)	4
View my health records, such as lab results	47.7% (31/65)	32.3% (21/65)	20.0% (13/65)	6
Track my medications	58.2% (39/67)	23.9% (16/67)	17.9% (12/67)	4
Track my oxygen levels	65.7% (44/67)	14.9% (10/67)	19.4% (13/67)	4
Track other health measures (e.g., heart rate, blood pressure)	39.7% (27/68)	27.9% (19/68)	32.4% (22/68)	3
Track my daily activity (e.g., daily steps)	67.2% (43/64)	15.6% (10/64)	17.2% (11/64)	7

Table 3: Frequency of Activities of Digital Technologies

Digital Technology Activity	Frequency Reported			
	Not at all	A little bit/Somewhat	Quite a bit/ Very much	Did not answer
Sending and receiving emails	16.9% (12/71)	21.1% (15/71)	62.0% (44/71)	0
Sending and receiving text messages	34.3% (24/70)	18.6% (13/70)	47.1% (33/70)	1
Participating in group text chats	82.6% (57/69)	13% (9/69)	4.3% (3/69)	2
Video calls (e.g., Skype, Zoom, FaceTime)	69.6% (48/69)	17.4% (12/69)	13.0% (9/69)	2
Participating in online support groups that were not related to COPD	78.6% (55/70)	20% (14/70)	1.4% (1/70)	1
Downloading apps	50% (33/66)	39.4% (26/66)	10.6% (7/66)	5
Searching on the internet/ Googling for information	16.9% (12/71)	36.7% (26/71)	46.5% (33/71)	0
Reading posts on social media sites, blogs or online forums	43.3% (29/67)	29.9% (20/67)	26.9% (18/67)	4
Posting on social media sites, blogs or online forums	75.4% (52/69)	21.7% (15/69)	2.9% (2/69)	2
Writing about my life experiences	75.4% (52/69)	18.8% (13/69)	5.8% (4/69)	2
Ancestry	59.2% (42/71)	25.4% (18/71)	15.5% (11/71)	0
Watching online videos (e.g., You-tube)	35.7% (25/70)	41.4% (29/70)	22.9% (16/70)	1
Listening to audio recordings or podcasts	58.0% (40/69)	27.5% (19/69)	14.5% (10/69)	2
Booking appointments online	56.3% (40/71)	28.2% (20/71)	15.5% (11/71)	0
Playing video games online	59.2% (42/71)	21.1% (15/71)	19.7% (14/71)	0
Completing surveys online	50.7% (36/71)	42.2% (30/71)	7.0% (5/71)	0
Online banking	44.3% (31/70)	12.9 (9/70)	42.9% (30/70)	1
Online dating	92.6% (63/68)	4.4% (3/68)	2.9% (2/68)	3
Online volunteering	94.3% (66/70)	4.3% (3/70)	1.4% (1/70)	1
