# Supplementary material for: From illness management to quality of life: rethinking consumer health informatics opportunities for progressive, potentially fatal illnesses
Source: J Am Med Inform Assoc. 2023 Dec 22;31(3):674–91. doi: 10.1093/jamia/ocad234 (PMC10873853; doi:10.1093/jamia/ocad234)
Supplement: ocad234_Supplementary_Data [file ocad234_supplementary_data.zip › ocad234_Supplementary_Data/Multimedia Appendix SC_Qualitative Data from Participants Living with a ....pdf]

Multimedia Appendix SC: Qualitative Data from Participants Living with a Progressive, Potentially Fatal Lung Condition Other than COPD

Experiences in being identified with the COPD community

People always assume, they see me walking around with an oxygen thingy and they assume it's COPD, but it's not. [PA01, Stage-1]

I felt maybe I was over stepping my bounds by pushing the ARDS stuff when you were concentrating on COPD.... I mean there's nobody else, no other groups around so… [the COPD support group] is better than nothing… And that's why I'm an odd ball. And I don't know of anybody else with it. [PA21, Stage-3]

I was diagnosed with COPD... then they did not diagnose it [as] COPD ... then eight years later I end[ed] up at emerge at another hospital, the doctors like COPD. I'm like, no I don't have COPD. She's says, yay you do. So then I went back to my respirologist, and she's like no you don't, you have chronic lung inflammation... like sixth visit to emerge... and the guys like "yes, something's going on, we're gonna get to the bottom of this". He sent me to the urgent care clinic, [I] go through all these tests over like three months, had a lung scan everything and the doctors ... [says] you have "sarcoidosis". [PA07, Stage-1]

Magnifying the isolation

I struggle with depression about it, ah [breath], I ... I've really noticed that my friends do not call me as often. I don't think it is because I have turned into a nasty person, or anything. As somebody explained to me, they are afraid to be around me in case something happens. Which is understandable, it's human nature. [PA01, Stage-1]

The connections I do have…, I didn't disclose how sick I was because I didn't wanna be a burden. Plus, I didn't wanna know, everyone to know how sick I was. [PA07, Stage-1]

Digital technologies for social connectedness

Technology for social connectedness

I do find that current technology is more isolating, okay [breath], uhm. People wanna text. No, 'A' I wanna to talk to you, if I can't actually see you. It's like my daughter, if I can't see her and give her a big hug and smell her skin and feel her hair, okay, know that it's her, then at least I want to talk to her. [PA01, Stage-1]

Virtual family reunions

In April they got me going with Zoom, which as I said I'm not very fussy about, but my daughter-in-law sent me an email saying connect in at this time. The reason for that was all of our family got together because most of them are, all of them are in [another province]... and it was my birthday so they all got together and talked to me on [Zoom] which was kind of nice. [PA21, Stage-3]

Loss of their connections

I [used to] go to my events like [the COPD group and my hobby group], and a few other things, but … and it doesn't bother me. I shouldn't say it doesn't bother me, it bothers me… I didn't recognize the COVID as a component of that. [PA21, Stage-3]

"Lack of information" on treatments

Lack of resources and information about their illness

It's scary. I was super isolated, plus I didn't know what I had and so you know. Search-searching for a support group? For what?... Very frustrating. [PA07, Stage-1]

Well I haven't really found anything to support because I haven't really looked in a while, I must admit. I did look and there wasn't very much on at all available so I just basically said ... I don't need to keep looking for it. I will one day again go back and see if I can find anything else but I haven't really found anything. [PA21, Stage-3]

Lack of information on recent medication and treatment advancements

As far as solving the ARDS component of it, there is nothing to solve, as far as they're concerned. So that's the difference why it's an odd ball thing, because COPD you get something to relieve that but I can't get anything to relieve mine. [PA21, Stage-3]

Follow-up and the need to advocate

I have to live on the mainland for at three months after surgery and if things don't go well, maybe six months. I'm going to be called upon to spend out of my own pocket twenty to fifty thousand dollars, okay for my [lung transplant] recovery time... I'm actually debating taking it up with my MLA. Because this is not right. [PA01, Stage-1]

Information seeking

I've worked online what the reputable sites... WebM... I'll usually go for the Mayo clinic stuff, and, anything research based, I really am very interested in reading any of the research that's new. [PA07, Stage-1]

Well I haven't really found anything to support because I haven't really looked in a while, I must admit. I did look and there wasn't very much on at all available so I just basically said "ok", you know, I don't need to keep looking for it. [PA21, Stage-3]

I do various research online when I am given drugs [and] when I look at survival expectancies. [PA01, Stage-1]

Self Management

Monitoring and tracking about their illness

No, no, there was some [health apps] and I wasn't solely enthused with them, yea, most of them are how many steps do you take in a day, well, I don't care… I don't [track my health information], I mean there's days when I feel good, there's days when I feel bad. [PA21, Stage-3]

Intergenerational connections

Connections to grandchildren

A lot of the kids, my grandkids and kids are on Facebook which, anyway, that's Facebook. Or Instagram, or you know those different ones. [PA21, Stage-3]

Carrying on tradition, leaving a legacy and passing on memories

My mom wrote a life story and I'm using [my tablet] very sporadically to write down memories... I'm writing down memories, that hopefully at one point I will knit them altogether into a life story. [PA01, Stage-1]

I used a program called Family Tree Maker on my computer. I've stopped doing so much because we have so many grandchildren and great grandchildren between our siblings, that I can't keep track of them all so I only right now am trying to keep track of my family only. [PA21, Stage-3]
